# Selective Sweeps Lead to Evolutionary Success in an Amazonian Hyperdominant Palm

**DOI:** 10.3389/fgene.2020.596662

**Published:** 2020-12-23

**Authors:** Warita A. Melo, Lucas D. Vieira, Evandro Novaes, Christine D. Bacon, Rosane G. Collevatti

**Affiliations:** ^1^Laboratório de Genética & Biodiversidade, Instituto de Ciências Biológicas, Universidade Federal de Goiás, Goiânia, Brazil; ^2^Departamento de Biologia, Universidade Federal de Lavras, Lavras, Brazil; ^3^Department of Biological and Environmental Sciences, University of Gothenburg, Gothenburg, Sweden; ^4^Gothenburg Global Biodiversity Centre, Gothenburg, Sweden

**Keywords:** Population Genomics, neutral evolution, *Mauritia flexuosa*, Arecaceae, adaptation, target sequence capture

## Abstract

Despite the global importance of tropical ecosystems, few studies have identified how natural selection has shaped their megadiversity. Here, we test for the role of adaptation in the evolutionary success of the widespread, highly abundant Neotropical palm *Mauritia flexuosa.* We used a genome scan framework, sampling 16,262 single-nucleotide polymorphisms (SNPs) with target sequence capture in 264 individuals from 22 populations in rainforest and savanna ecosystems. We identified outlier loci as well as signal of adaptation using Bayesian correlations of allele frequency with environmental variables and detected both selective sweeps and genetic hitchhiking events. Functional annotation of SNPs with selection footprints identified loci affecting genes related to adaptation to environmental stress, plant development, and primary metabolic processes. The strong differences in climatic and soil variables between ecosystems matched the high differentiation and low admixture in population Bayesian clustering. Further, we found only small differences in allele frequency distribution in loci putatively under selection among widespread populations from different ecosystems, with fixation of a single allele in most populations. Taken together, our results indicate that adaptive selective sweeps related to environmental stress shaped the spatial pattern of genetic diversity in *M. flexuosa*, leading to high similarity in allele frequency among populations from different ecosystems.

## Introduction

The geographic distribution of genetic diversity results from the interplay between demographic forces (i.e., genetic drift and gene flow), mutation, recombination, and natural selection (Wright, 1931). The study of adaptation to environmental conditions has led to an understanding of how species cope with climate and landscape changes (e.g., Evans et al., 2014; Gratani, 2014; Yeaman et al., 2014; Hornoy et al., 2015). Genome scan approaches have contributed to the understanding of adaptive responses (Savolainen et al., 2013; Tiffin and Ross-Ibarra, 2014), especially because local adaptation may affect genetic variation at specific loci (Smith and Haigh, 1974; Kaplan et al., 1989; Orr, 1998; Kawecki and Ebert, 2004), allowing for the identification of genomic regions that are tightly linked to or directly under natural selection. However, it is important to note that most adaptive traits are polygenic with large number of alleles with small effects (McKay and Latta, 2002; Pritchard and Di Rienzo, 2010). In these traits, selection may cause only small changes in allele frequencies, and the response to selection may be the outcome of allelic covariances among loci (McKay and Latta, 2002; Pritchard and Di Rienzo, 2010; Le Corre and Kremer, 2012).

Evidence of local adaptation has been identified in plants from temperate climates (Parchman et al., 2012; Yeaman et al., 2014, 2016; Hornoy et al., 2015; Jordan et al., 2017), yet only recently have these approaches been applied to tropical species (e.g., Lanes et al., 2018; Collevatti et al., 2019; Brousseau et al., 2020). Despite recent advances, the detection of selection footprints using genome scans is still limited because of demographic history, which can constrain the detection of other types of selective forces, such as background, balancing, or purifying selection (Otto, 2000; Vitti et al., 2013). The detection of selection footprints may also be hindered by range shifts due to paleoclimatic changes, causing incomplete lineage sorting and high genetic differentiation (Collevatti et al., 2019). Thus, disentangling the effects of demographic history from selection in extant genetic diversity is important to better understand the evolution of species distributions, but remains a challenge.

Target sequence capture has been increasingly used to genotype large numbers of markers because it significantly reduces costs and effort compared with whole-genome sequencing and can be used to study the role of evolution and ecology at the genome-wide scale (Jones and Good, 2016; Silva-Junior et al., 2018; Andermann et al., 2019). In plants, the method has been applied successfully to robustly estimate population genetic parameters in groups such as maize (Fu et al., 2010), pine [*Pinus taeda*, (Neves et al., 2013); *Pinus albicaulis* (Syring et al., 2016)], poplars (Zhou and Holliday, 2012), and tropical trees and palms (e.g., Collevatti et al., 2019; Loiseau et al., 2019).

**FIGURE 1:**
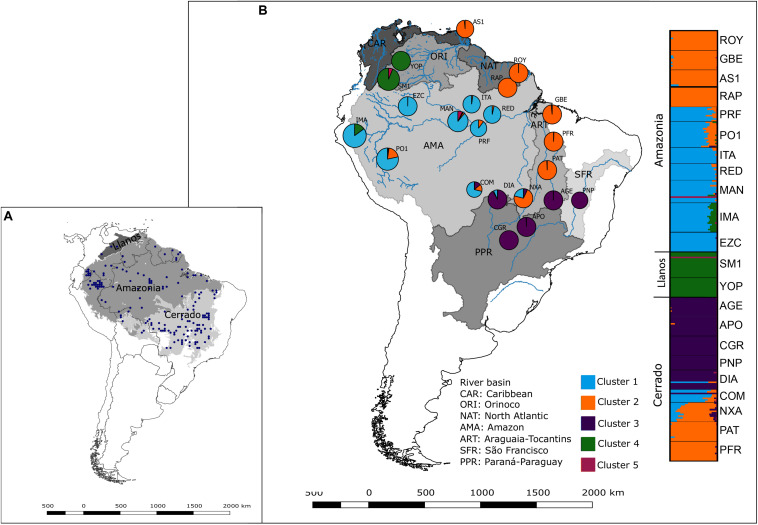
**(A)** Geographic distribution of Mauritia flexuosa in South America. **(B)** Geographic distribution of the 22 *Mauritia flexuosa* populations sampled for genetic analyses and Bayesian clustering of individuals based on 3,470 putatively neutral SNP loci. Each color represents an inferred genetic cluster (*K* = 5). The size of cluster chart section represents population coancestry for each cluster. The plot shows the best *K* = 5 found in six from 10 runs. The inset map shows the geographic distribution of *M. flexuosa* based on the occurrence records (black dots) from the Global Biodiversity Information Facility (https://www.gbif.org). The shape file of river basins was obtained from *HydroBASINS* v. 1c (Lehner and Grill, 2013). Details on the sampled populations are provided in Supplementary Table S1 in Supplementary Appendix 1.

Palms have high levels of genetic and phenotypic variation and a long and rich fossil history (Baker and Dransfield, 2016). The family has a wide distribution across large geographic areas and latitudinal ranges, but climatic variables cause palms to be spatially restricted along temperature and other geographic and environmental gradients (Eiserhardt et al., 2013; Freitas et al., 2019). *Mauritia flexuosa* L. f. (Arecaceae) is one of the most hyperdominant species in Amazonia and is common in swampy environments (Rull, 1998; Ter Steege et al., 2013; Rull and Montoya, 2014). It has a large geographic range across different ecosystems (Figure 1): in lowland tropical forest in the Amazon and Orinoco Basins and the Guiana Shields, and in savannas in the Llanos and the Brazilian Cerrado.

The high abundance and wide geographic distribution of *M. flexuosa* raise the question of how the species reached such level of evolutionary success. The species is dioecious and wind-pollinated (Khorsand and Koptur, 2013). Fossil records and phylogeographic analyses of *M. flexuosa* show a capacity for resilience to climate change, at least through Quaternary range shifts (e.g., de Lima et al., 2014; Melo et al., 2018). Moreover, the species has high genetic differentiation among populations from different ecosystems and river basins as shown by chloroplast (de Lima et al., 2014) and microsatellite loci (Melo et al., 2018; Sander et al., 2018), suggesting that genetic drift may shape the current distribution of genetic variation in *M. flexuosa*. A genome-wide scan allows for a comprehensive evaluation of the relative roles of genetic drift and selection in the spatial distribution of genetic diversity and provides an unprecedented window into the evolutionary success of this iconic Neotropical palm species.

Here, we test between local adaptation and genetic drift as a driver of the evolutionary success characterizing the high abundance and broad geographic range of the hyperdominant species *M. flexuosa*. We identify local adaptation through detection of outlier loci and correlation of allele frequencies with environmental variables that indicate ecosystem-based differences among populations. To identify selective sweeps and genetic hitchhiking events, we used likelihood ratio tests. For these tests, we sampled populations in three different ecosystems across its geographic distribution, genotyping 16,262 single-nucleotide polymorphisms (SNPs) with target sequence capture approach. Coalescent simulations were used to reconstruct the demographic history of the species based on putatively neutral SNPs in an attempt to disentangle the effects of demography (genetic drift and gene flow) and natural selection on the distribution of genetic diversity. Because of the contrasting environmental conditions of the different ecosystems, i.e., tropical rain forest and seasonal savanna, we expect to find local adaptation and high differentiation in allele frequencies among ecosystems.

## Materials and Methods

### Population Sampling

We sampled 264 individuals from 22 populations of *M. flexuosa* throughout its geographic range (Figure 1 and Supplementary Table S1 in Supplementary Appendix 1). Populations were sampled in Amazonia (11 populations), Llanos (two), and Cerrado (nine) ecosystems across different river basins: eight populations from the Amazon basin, one in the Caribbean, two in the North Atlantic, two in the Orinoco, three in the Paraná-Paraguay, one in the São Francisco, and five in the Araguaia-Tocantins (Figure 1; Supplementary Table S1 in Supplementary Appendix 1). Special authorization in Brazil was not necessary for sampling, because we sampled outside protected reserves. Permits for leaf collection were granted from Trinidad through the University of the West Indies and the Department of Natural Resources and the Environment to S. Federman; from Bolivia by the Ministry of Environment and Water to the Bolivian National Herbarium for carrying out research under the PALMS 2009–2013 project led by H. Balslev; from Colombia by the Ministry of the Environment and Sustainable Development under the Autoridad Nacional de Licencias Ambientales (Resolution 0790) to CES University in collaboration with MJ Sanín Perez; and from Peru through the Instituto Nacional de Recursos Naturales of the Ministry of Agriculture to H. Balslev (690-2007-INRENA-IFFS-DCB). In Brazil, vouchers where collected in each population and compared to exsiccate UB8821 from UNB herbarium (Universidade de Brasilia, Brasília, DF, Brazil). A shape file of river basins was obtained from HydroBASINS v. 1c (Lehner and Grill, 2013). Leaves of adults were collected, and geographic coordinates were taken for each individual.

### SNP Genotyping

We used a set of 2,909 120-mer probe sequences targeting single copy loci derived from *Cocos nucifera*, *Elaeis guineensis*, *Nypa fruticans*, *Phoenix dactylifera*, and *Sabal bermudana* covering 169 single-copy nuclear genes for target sequence capture (Heyduk et al., 2016). *M. flexuosa* has 2*n* = 30 and genome size 1C = 4.7 pg (Röser et al., 1997). We extracted the DNA using Qiagen DNeasy Plant Mini kit (Qiagen, DK). Targeted DNA enrichment, capture, and sequencing were carried out by RAPiD Genomics LLC (Gainesville, FL, United States) using the SureSelectXT (Agilent Technologies, CA, United States) enrichment system and Illumina DNA sequencing on a HiSeq2000 instrument (Illumina, CA, United States). Genomic DNA libraries were prepared using the Agilent protocols, including DNA shearing followed by ends repair, 3′-end adenylation, adaptor ligation, and amplification following Neves et al., 2013. Libraries were pooled in equimolar amounts and sequenced in two lanes of an Illumina HiSeq2000 instrument (Illumina, CA, United States), 2 × 101-bp mode.

Sequencing reads were trimmed for adapters using Trimmomatic v. 0.39 (Bolger et al., 2014). Stretches of low-quality sequences were removed from reads using the option SLIDINGWINDOW: 4:20, and sequences smaller than 32 bp were discarded. Reads were aligned to the probe sequences with BWA v. 0.5.9 (Li and Durbin, 2009) using default settings in the BWA-backtrack algorithm. These steps were performed in a grid computing cluster software system using a pipeline available from the International Cassava Genetic Map Consortium (Alaba et al., 2015). SNP calling was performed using FreeBayes v. 1.1.0 (Garrison and Marth, 2012). FreeBayes genotyped biallelic SNPs only in the target region, using the following options:–theta 0.01–ploidy 2–min-alternate-fraction 0.2–min-alternate-count 2–min-coverage 8–genotype-qualities–min-mapping-quality 10–min-base-quality 20–report-genotype-likelihood-max–no-indels–no-mnps–no-complex. Data were finally filtered using VCFtools v. 0.1.12b (Danecek et al., 2011) with the following parameters:–max-missing 0.6–mac 2—minGQ 20–minDP 3.

### Genome-Wide Diversity and Genetic Structure

To characterize genome-wide diversity, we calculated the density of SNPs across all probes using a bin size of 100 bp, allele frequencies, and the percentage of missing data. We also inferred the ratio of transitions to transversions substitutions (Ts/Tv) and expected heterozygosity using *VCFtools*.

To characterize population genetic diversity across all SNP loci, we estimated expected heterozygosity under Hardy–Weinberg equilibrium (*He*) and inbreeding coefficient (*f*) using Arlequin v. 3.5 (Excoffier and Lischer, 2010). We used a hierarchical analysis of molecular variance (AMOVA) implemented in Arlequin to estimate the genetic differentiation between ecosystems (*F*_*CT*_) and among populations within ecosystems (*F*_*SC*_). We also analyzed the differentiation among river basins (*F*_*CT*_), among populations within river basins (*F*_*SC*_), and inbreeding coefficient (*F*_*IS*_). Significance levels of 0.05 for each estimate were determined with 10,000 permutations.

Further, we estimated genetic diversity and analyzed genetic structure in a subset of more stringently cleaned, and linkage disequilibrium (LD) pruned loci. We used PLINK 1.9 (Chang et al., 2015) to eliminate loci with more than 15% of missing data (—geno 0.15) and a minor allele frequency (MAF) lower than 1% (—maf 0.01). The LD pruning was also performed with PLINK with sliding windows of 50 SNPs, removing associated loci (*R*^2^ > 0.30) using the option —indep-pairwise 50 5 0.3. The list of cleaned and LD-pruned SNPs was checked for SNPs with positive selection signal, and none were identified, based on Outflank and Bayenv2 results. Therefore, this list of potentially neutral and high-quality SNPs was used to estimate genetic diversity and to perform an AMOVA. Finally, we used Bayesian clustering implemented in fastSTRUCTURE software (Raj et al., 2014) to identify the most likely number of genetic clusters among *M. flexuosa* samples. The analysis was performed with number of groups *K* ranging from 2 to 22. We assessed the most likely number of clusters supported by the data in Structure Selector (Li and Liu, 2018), with a threshold of 0.5, following (Puechmaille, 2016). To generate output files, we used CLUMPAK v. 1.1 (Kopelman et al., 2015). For this set of potentially neutral loci, we analyzed whether populations are isolated by distance using a linear regression of linearized *F*_*ST*_ on the logarithm of geodesic geographic distance. We also performed an autocorrelation analyses using Moran’s *I*, implemented in the software SAM (Spatial Analysis in Macroecology; Rangel et al., 2010), to understand the effect of spatial scale in genetic differentiation.

### Simulation of Demographic History

To test whether demographic dynamics in the Quaternary were responsible for the distribution of allele frequencies in extant populations of *M. flexuosa*, we modeled the demographic history and performed simulations for 3,500 potentially neutral loci. We simulated two demographic scenarios derived from ecological niche modeling (see de Lima et al., 2014; Melo et al., 2018): “Range Expansion” (smaller range size at the LGM compared to present day) and “Range Stability” (similar range size at the LGM and present day). We also simulated the scenario of “Multiple Refugia” (retraction of savanna-like vegetation during glacial periods leading to many refugia of different effective sizes) derived from paleovegetation reconstruction and fossil record. We simulated 22 demes using the software DIYABC v. 2.1 (Cornuet et al., 2014). For model calibration, we used the demographic parameters estimated using coalescent analyses following Melo et al. (2018). Population dynamics were simulated backward (see Supplementary Figure S1 in Supplementary Appendix 2) from the present (*t* = 0) to *t* = 525 generations ago (at 21 ka, using a generation time of 40 years; Melo et al., 2018), with effective size drawn from a uniform distribution with minimum Ne = 300 and maximum Ne = 3,000 (Supplementary Figure S1 in Supplementary Appendix 2).

Mean number of alleles, mean expected heterozygosity, and *F*_*ST*_ were inferred from 600,000 simulations and compared with the observed values and the relative fits of the models using approximate Bayesian computation (Cornuet et al., 2014), also implemented in DIYABC. We used the relative proportion of each scenario in the simulated data set closest to the observed data set (hereafter, direct approach) and the logistic regression (hereafter, logistic approach) of each scenario probability on the deviation between simulated and observed summary statistics (Cornuet et al., 2008).

### Genome Scans for Selection Footprints

To test for local adaptation, we used three different genome scan approaches to identify loci under selection. First, OutFLANK (Whitlock and Lotterhos, 2015) was used based on the expected χ^2^ distribution of *F*_*ST*_ in the absence of selection. To generate the distributions, we trimmed the *F*_*ST*_ distribution at 5% and 10% and used a minimum expected heterozygosity of 0.10 and a false discovery rate <0.05.

Secondly, we used a Bayesian framework implemented in Bayenv2 (Coop et al., 2010; Günther and Coop, 2013) to identify local adaptation by estimating linear correlations between allele frequencies and environmental variables, while controlling for relationships among populations. Four bioclimatic variables from the WorldClim Global Climate database^1^ with a spatial resolution of 30” (0.93 × 0.93 = 0.86 km^2^ at the equator) were obtained for the 22 populations sampled. The four bioclimatic variables were identified by low collinearity in factorial analysis with Varimax rotation. These four variables explained 90.4% of the total environmental variation among the 22 populations (Supplementary Table S2 in Supplementary Appendix 1). The selected variables were as follows: annual mean temperature, mean diurnal range, precipitation of driest month, and precipitation of the wettest quarter.

We also obtained soil data related to soil fertility from the Harmonized World Soil Database v. 1.2^2^. Varimax factorial analysis selected four soil variables that explained 77.5% of the variation among populations (Supplementary Table S3 in Supplementary Appendix 1): topsoil salinity, topsoil organic carbon, topsoil silt fraction, and topsoil reference bulk density. Loci with adaptive selection signal were selected following Günther and Coop, 2013: high Bayes factor (>100.0), i.e., the likelihood ratio of the probability of the linear relationship hypothesis (between allele frequency and environmental variable) and the null hypothesis (no linear relationship) given the data and a Spearman correlation coefficient | ***ρ*** | > 0.15. These values were in the 0.1% quantile of the distribution of the data.

For the loci under selection, we calculated the shift in allele frequency between pairs of populations, i.e., the difference between the allele frequency of the population with highest and lowest value for each climatic and soil variable and for each SNP locus. For each variable, we obtained a vector of differences in allele frequency for the loci, and then, we plotted the density function using the function density implemented in the stat package of R v. 3.6.1 (R Core Team, 2016).

Finally, SweepFinder2 v. 1.0 (Degiorgio et al., 2016) was used to detect selective sweeps and genetic hitchhiking based on deviations of a neutral null hypothesis. We applied the composite likelihood ratio (CLR) to identify possible location of recent selective sweeps in each of the 22 populations, following Nielsen, 2005. We used only contigs containing loci with evidence of adaptive selection identified in Bayenv2. We assumed unknown polarity and considered that the lower frequency allele was the derived state. For each contig, the CLR function was calculated on a grid of 122 positions along the length of the probe sequence. The selective sweep CLR test statistic was the maximum composite likelihood value optimized over all possible positions, compared to the composite likelihood of the neutral null model as calculated by the program. We simulated new neutral data sets with population scaled mutation rate (θ) and population scaled recombination rate (ρ) typical for highly heterozygous tropical forest trees estimated from genome-wide data (Silva-Junior and Grattapaglia, 2015). We used *θ/bp* = 0.018 bp^–1^ and *p/bp* = 0.0011 bp^–1^, assuming homogenous rates among regions. Samples with the same number of individuals in the real populations and sequence length of scaffolds were generated under Wright–Fisher model using the software *ms* (Hudson, 2002) with 100 replicates per scaffold. For each replicate, multiple alignments were performed using MAFFT v. 7.130b (Katoh and Standley, 2013), and variant sites were detected across the samples using SNP sites (Page et al., 2016). The composite likelihood inference was repeated for each replicate in SweepFinder2 using the same grid size as for the real data. Composite likelihood values were collected from all the replicates across all positions along the length of the simulated sequences totaling 10.7 million data points. A cumulative histogram of the values was used to obtain the neutral composite likelihood threshold of 95% (Supplementary Figure S2 in Supplementary Appendix 2). The value of 1.22 was used as the significance cutoff. The probable locations of sweeps were taken as the maximum composite values above the significance cutoff calculated for each scaffold.

### Annotation of SNP Loci Under Selection

Putative SNP loci under selection were annotated against the genomes of Embryophytes of the Phytozome v. 12.1 (Goodstein et al., 2012) database^3^. The probe sequences were used in a BlastX, and in the few cases where no Blast hit was obtained, the *E. guineensis* and *M. flexuosa* transcriptomes were used (Supplementary Table S1 in Supplementary Appendix 3). Affected genes were analyzed with respect to their functional annotation in terms of Gene Ontology, PFAM, and PANTHER categories obtained from the Blast best hits (Ashburner et al., 2000).

## Results

### SNP Detection and Genotyping

Using target sequence capture, we obtained more than 37 Gbp from the 264 individuals. A total of 366,464,311 reads were sequenced, with a mean of 832,873 reads per individual. The sequencing generated 34,569 SNPs, which after data filtering resulted in 16,262 high-quality polymorphic SNPs, with an average call rate of 93.2%. The average per-sample call rate across all 264 individuals was 93.1%. The transitions-to-transversions ratio was 2.06. The average missing genotype per loci was 6.8% in the sample of 264 individuals, and 6.9% per individual sampled (Supplementary Figure S3 in Supplementary Appendix 2). The distribution of MAF showed the expected “L” shape distribution with a larger proportion of low frequency SNPs. The vast majority of the SNPs (82.2%) had MAF < 0.15, and the median MAF was equal to 0.079. The average read depth at SNPs coordinates across samples was 25.4*x* ± 34.5*x*, and the minimum depth of aligned reads to call a heterozygous genotype for a sample was three with the median equal to 26.

### Genome-Wide Diversity and Genetic Structure

The average SNP density was 1.06 SNP/100 bp (median = 1, min = 0.1, max = 4.20; Supplementary Figure S3 in Supplementary Appendix 2, Supplementary Table S2 in Supplementary Appendix 3). Genetic diversity was high in all populations (Table 1) and the total genetic diversity considering all the 16,262 SNPs (*He* = 0.338, SD = 0.174) was similar to the diversity obtained with only putatively neutral loci (*He* = 0.357, SD = 0.023).

**TABLE 1:** Genetic diversity in 22 populations of *Mauritia flexuosa*, based on all SNPs (16,262 SNPs) and on 3,479 potentially neutral SNPs.

				**All SNPs**				**Neutral SNPs**		
**Ecosystem**	**River basin**	**Population**	***n***	**No. loci**	***He* (SD)**	***Ho* (SD)**	***f***	***He* (SD)**	***Ho* (SD)**	***f***
Amazonia	Amazon	COM	8	5,322	0.345 (0.172)	0.502 (0.352)	−0.576	0.361 (0.164)	0.523 (0.344)	−0.509
		EZC	12	4,457	0.362 (0.169)	0.548 (0.354)	−0.614	0.347 (0.169)	0.515 (0.350)	−0.528
		IMA	18	5,447	0.311 (0.187)	0.470 (0.368)	−0.593	0.316 (0.178)	0.472 (0.355)	−0.523
		ITA	10	4,244	0.385 (0.162)	0.593 (0.350)	−0.622	0.363 (0.165)	0.542 (0.350)	−0.557
		MAN	14	6,264	0.296 (0.180)	0.397 (0.373)	−0.408	0.317 (0.173)	0.436 (0.354)	−0.418
		PO1	16	3,803	0.353 (0.176)	0.541 (0.367)	−0.658	0.342 (0.174)	0.537 (0.363)	−0.575
		PRF	9	4,091	0.396 (0.160)	0.608 (0.346)	−0.630	0.374 (0.164)	0.560 (0.348)	−0.569
		RED	10	4,096	0.392 (0.163)	0.611 (0.356)	−0.636	0.369 (0.166)	0.555 (0.357)	−0.579
		Mean	12.1	—	0.355	0.534	—	0.354	0.530	—
		SD	3.6	—	0.171	0.358	—	0.021	0.040	—
		Total	97	—	0.179 (0.199)	—	−0.599	0.228 (0.190)	—	−0.517
	Caribbean	AS1	10	3,496	0.392 (0.160)	0.615 (0.353)	−0.655	0.382 (0.161)	0.587 (0.351)	−0.614
	North Atlantic	RAP	12	3,578	0.372 (0.167)	0.588 (0.363)	−0.666	0.364 (0.166)	0.567 (0.359)	−0.618
		ROY	12	3,593	0.376 (0.167)	0.595 (0.363)	−0.659	0.367 (0.168)	0.569 (0.361)	−0.615
		Mean	12	—	0.374	0.592	—	0.365	0.568	—
		SD	0	—	0.167	0.363	—	0.001	0.569	—
		Total	24	—	0.335 (0.335)	—	−0.661	0.324 (0.181)	—	−0.592
Overall Amazonia		Mean	12.2	—	0.351	0.525	—	0.363	0.542	—
		SD	3.0	—	0.167	0.354	—	0.021	0.042	—
		Total	159	—	0.113 (0.171)	—	−0.509	0.218 (0.191)	—	−0.530
Lhanos	Orinoco	SM1	16	12,076	0.196 (0.146)	0.161 (0.307)	0.172*	0.286 (0.171)	0.344 (0.355)	−0.281
		YOP	12	4,123	0.381 (0.165)	0.597 (0.355)	−0.657	0.376 (0.160)	0.577 (0.349)	−0.587
		Mean	14	—	0.289	0.379	—	0.331	0.344	—
		SD	2.8	—	0.156	0.331	—	0.045	0.577	—
		Total	28	—	0.165 (0.162)	—	−0.119	0.261 (0.180)	—	0.367
Cerrado	Paraná-Paraguay	APO	12	4,194	0.326 (0.182)	0.497 (0.370)	−0.757	0.368 (0.171)	0.591 (0.362)	−0.709
		CGR	12	4,222	0.314 (0.190)	0.484 (0.383)	−0.630	0.348 (0.179)	0.545 (0.372)	−0.625
		DIA	12	5,726	0.299 (0.187)	0.448 (0.370)	−0.618	0.346 (0.176)	0.531 (0.367)	−0.570
		Mean	12	—	0.313	0.476	—	0.348	0.545	—
		SD	0	—	0.186	0.374	—	0.009	0.025	—
		Total	36	—	0.209 (0.196)	—	−0.696	0.257 (0.193)	—	−0.649
	São Francisco	PNP	9	3,436	0.380 (0.169)	0.598 (0.369)	−0.662	0.383 (0.162)	0.593 (0.359)	−0.639
	Araguaia-Tocantins	AGE	12	5,097	0.303 (0.187)	0.456 (0.366)	−0.649	0.352 (0.177)	0.550 (0.364)	−0.606
		GBE	12	5,613	0.294 (0.186)	0.439 (0.365)	−0.627	0.338 (0.177)	0.520 (0.365)	−0.583
		NXA	12	5,607	0.302 (0.186)	0.454 (0.366)	−0.629	0.341 (0.176)	0.526 (0.359)	−0.588
		PAT	12	4,040	0.337 (0.184)	0.527 (0.380)	−0.656	0.353 (0.175)	0.548 (0.366)	−0.611
		PFR	12	3,936	0.332 (0.184)	0.511 (0.368)	−0.810	0.384 (0.164)	0.619 (0.346)	−0.731
		Mean	12	—	0.314	0.477	—	0.352	0.548	—
		SD	0	—	0.185	0.369	—	0.016	0.035	—
		Total	60	—	0.176 (0.191)	—	−0.696	0.239 (0.191)	—	−0.65
Overall Cerrado		Mean	11.7	—	0.321	0.49	—	0.352	0.548	—
		SD	1	—	0.184	0.371	—	0.016	0.032	—
		Total	105	—	0.154 (0.188)	—	−0.685	0.225 (0.192)	—	−0.638
Overall ecosystems		Mean	12	—	0.338	0.511	—	0.357	0.546	—
		SD	2.4	—	0.174	0.361	—	0.023	0.057	—
		Total	264	—	0.107 (0.168)	—	−0.583	0.206 (0.184)	—	−0.559

*N, number of genotyped individuals; N loci, number of polymorphic loci; He, mean expected heterozygosity under Hardy–Weinberg equilibrium; Ho, mean observed heterozygosity; *f*, inbreeding coefficient (all values are significantly different from zero, *p* < 0.05); SD, standard deviation.*

To perform a genetic structure analysis for putatively neutral loci, the SNPs were further cleaned to remove loci in LD. The 3,479 loci after LD pruning had no selection signal. Therefore, these neutral LD pruned SNPs were used in a Bayesian clustering method, which supported six genetic clusters (*K* = 6) with low admixture in populations from different ecosystems (Figure 1 and Supplementary Figures S4, S5 in Supplementary Appendix 2; Supplementary Tables S4–S6 in Supplementary Appendix 1). However, no individual was assigned to cluster 6 with coancestry coefficient *Q* > 0.001, being impossible to display in a map due to the relative scale (Supplementary Table S4 in Supplementary Appendix 1). Thus, we chose the partition of *K* = 5 (Figure 1 and Supplementary Tables S5, S6 in Supplementary Appendix 1).

The 22 populations showed low and significant genetic differentiation for the 16,262 SNPs (*F*_*ST*_ = 0.070, *p* < 0.001) and negative and significant inbreeding coefficient (*F*_*IS*_ = −0.583, *p* < 0.001), showing higher frequency of mating between unrelated individuals than expected at random, as predicted for a dioecious species. Genetic differentiation was higher for the 3,470 putatively neutral loci (*F*_*ST*_ = 0.104, *p* < 0.001), and the inbreeding coefficient was significant (*F*_*IS*_ = −0.559, *p* < 0.001). Hierarchical AMOVA showed low, but significant differentiation among ecosystems (Amazonian, Llanos, and Cerrado), for the entire dataset (*F*_*CT*_ = 0.015, *p* < 0.001) and for the 3,470 putatively neutral, LD-pruned loci (*F*_*CT*_ = 0.063, *p* < 0.001). Differentiation among populations within ecosystems was low for the entire dataset (*F*_*SC*_ = 0.064, *p* < 0.001) and for the putatively neutral loci (*F*_*SC*_ = 0.067, *p* < 0.001). Differentiations among river basins (*F*_*CT*_ = 0.055, *p* < 0.001) and among populations within river basins (*F*_*SC*_ = 0.028, *p* < 0.001) were low but significant for the entire dataset, as well as for the 3,470 putatively neutral, LD-pruned loci (*F*_*CT*_ = 0.080, *p* < 0.001; *F*_*SC*_ = 0.041, *p* < 0.001). Genetic differentiation among pairs of populations was significantly correlated to geographical distance (*r*^2^ = 0.290, *p* < 0.001, Supplementary Figure S6A in Supplementary Appendix 2). Autocorrelation analysis showed a significant relationship between genetic differentiation and geographical distance for populations up to 1,500 km (Supplementary Figure S6B in Supplementary Appendix 2).

### Simulation of Demographic History

The demographic simulations using putatively neutral loci, to analyze the role of genetic drift in shaping the spatial distribution of *M. flexuosa* genetic diversity, supported a scenario of “Multiple Refugia” as the most probable predictor of the observed genetic parameters using the logistic approach (Supplementary Figure S7 in Supplementary Appendix 2; Supplementary Table S7 in Supplementary Appendix 1). Using the direct approach, we found little variation in likelihood among the different demographic scenarios tested (Supplementary Figure S6 in Supplementary Appendix 2).

### Genome Scans for Selection Footprints

The OutFLANK genome scan detected 45 outlier loci (Supplementary Table S8 in Supplementary Appendix 1, Supplementary Figures S8, S9 in Supplementary Appendix 2, and Supplementary Table S3 in Supplementary Appendix 3). These outlier loci had *F*_*ST*_ values ranging from 0.77 to 1.00 (Supplementary Figures S8, 9 in Supplementary Appendix 2).

**FIGURE 2:**
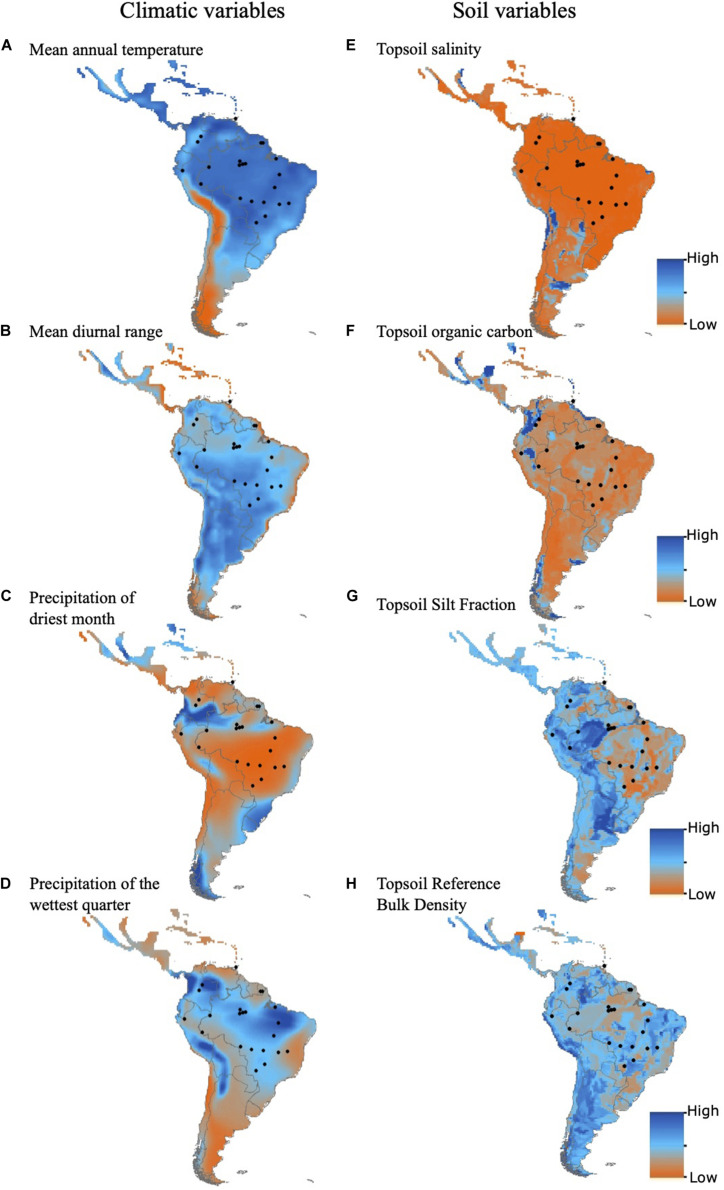
Geographic space of climatic and soil variables across the Neotropics. The geographic distribution of the *Mauritia flexuosa* populations sampled here is shown with black dots. **(A)** Annual mean temperature. **(B)** Mean diurnal range. **(C)** Precipitation of driest month. **(D)** Precipitation of the wettest quarter. **(E)** Topsoil salinity. **(F)** Topsoil organic carbon. **(G)** Topsoil silt fraction. **(H)** Top soil reference bulk density. The bioclimatic variables were obtained from the WorldClim Global Climate Bioclim database (www.worldclim.org/bioclim). Soil data were obtained from the Harmonized World Soil Database version 1.2 (FAO/IIASA/ISRIC/ISS-CAS/JRC 2009, available at http://www.fao.org/docrep/018/aq361e/aq361e.pdf).

**FIGURE 3:**
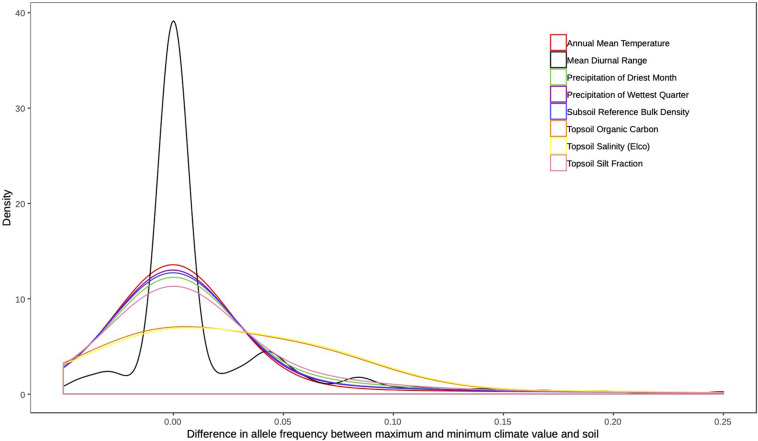
Comparison of the distributions of shifts in allele frequency for the 41 SNPs with selection signatures with the highest correlation with environmental variables. A shift is defined as the difference between the allele frequencies of the population with the highest and the lowest value for each climatic and soil variable. The density function was obtained for the vector of differences in allele frequency for the loci and calculated using the function *density* implemented in the *stat* package in R 3.6.0.

Bayenv2 identified outlier loci with high correlation to climatic variables (17 loci; Supplementary Table S9 in Supplementary Appendix 1, Supplementary Figure S10 in Supplementary Appendix 2, and Supplementary Table S4 in Supplementary Appendix 3) and to soil variables (24 loci; Supplementary Table S10 in Supplementary Appendix 1, Supplementary Figure S11 in Supplementary Appendix 2, and Supplementary Table S5 in Appendix 3). Correlation coefficients between allele frequency and the environmental variables ranged between ± 0.50 (Supplementary Figures S10, S11 in Supplementary Appendix 2). Climatic and soil variables varied among populations (Figure 2, Supplementary Table S11 in Supplementary Appendix 1), but did not show a clinal spatial pattern (Figure 2). Most loci under selection had small differences in allele frequency between populations with different climatic and soil conditions (Figure 3).

**FIGURE 4:**
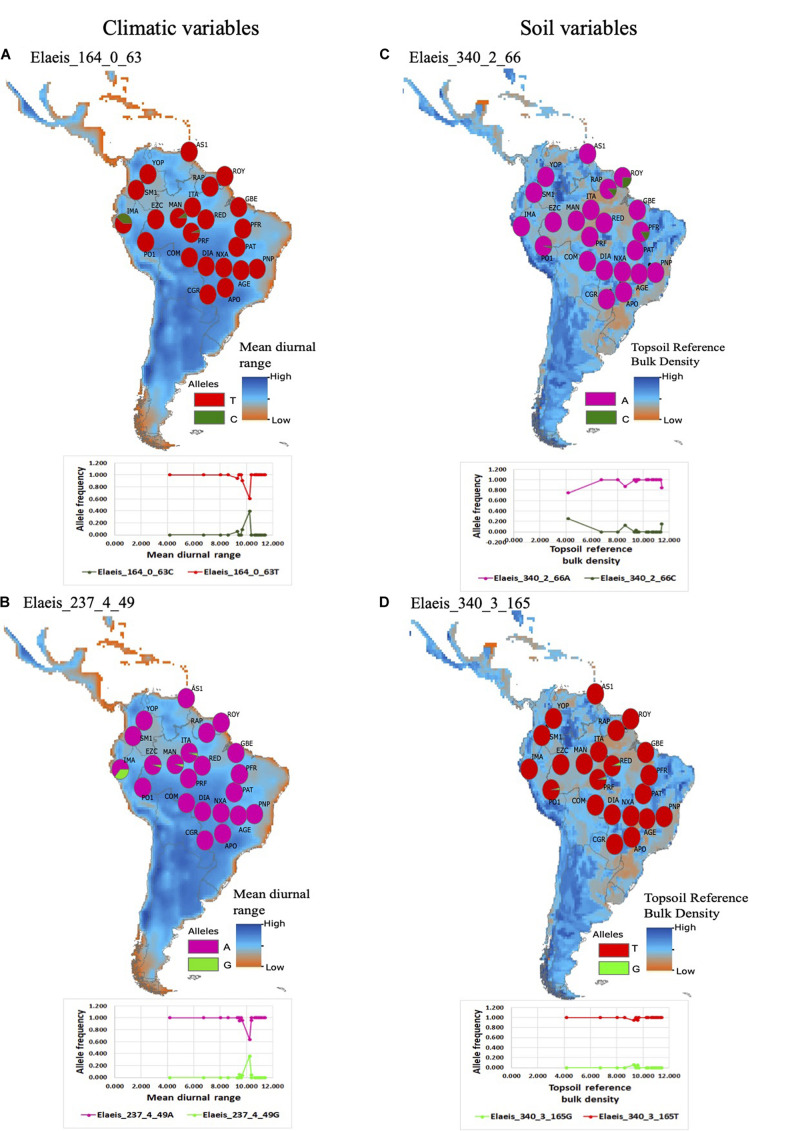
Geographic distribution of allele frequencies in climatic and soil geographic space (maps) for SNPs with under adaptive selection evidenced by Bayenv2 analysis and relationship between allele frequency and climatic and soil variables (plots below the maps). **(A)** Allele frequency for the SNP loci affecting the Mon1 trafficking protein gene correlated to mean diurnal range. **(B)** Allele frequency for the SNP loci affecting the Rab protein gene correlated to mean diurnal range. **(C)** Allele frequency for the SNP loci affecting the magnesium transporter MRS2-4 protein gene correlated to topsoil reference bulk density. **(D)** Allele frequency for the SNP loci affecting the magnesium transporter MRS2-4 protein gene correlated to topsoil reference bulk density.

We detected hard selective sweeps within 28 of the 41 contigs identified from Bayenv2 (Supplementary Table S12 in Supplementary Appendix 1). These 28 contigs contained 134 positions that were likely targets of selective sweeps (Supplementary Table S12 in Supplementary Appendix 1). Most populations were fixed for the same allele with selection signal (Figure 4, see also Supplementary Figures S12, S13 in Supplementary Appendix 2).

### Functional Annotation of SNPs With Selection Signal

Functional annotation indicated that the 45 outlier SNPs identified by OutFLANK are located in nine genes (Supplementary Table S8 in Supplementary Appendix 1). These genes are related to diverse biological processes, including housekeeping genes such as ribonuclease, ATP synthetase, Rab GDP dissociation inhibitors, and the GTPase family.

The 17 loci correlated to climatic variables are located in 16 genes (Supplementary Table S9 in Supplementary Appendix 1, Supplementary Table S6 in Supplementary Appendix 3), and the 24 loci correlated to soil variables affected 22 genes (Supplementary Table S10 in Supplementary Appendix 1, Supplementary Table S7 in Supplementary Appendix 3). The 38 genes with putative signals of adaptive selection are involved in a wide spectrum of biological processes such as ATP synthesis, DNA replication, mitotic checkpoint, and transcription factors (elongator acetyltransferase complex-ELP). Additionally, we found SNPs correlated to soil variables potentially affecting magnesium (Mg) transport (Mg transporter mrs2-4–related gene) and genes related to NAD(P) activity (Supplementary Tables S9, S10 in Supplementary Appendix 1).

SweepFinder2 analysis detected 28 SNP loci with selective sweeps signal located within 25 genes (Supplementary Table S12 in Supplementary Appendix 1). As expected, the list of genes is similar to and nested within those obtained with the Bayenv2 analyses. These include genes involved in cellular metabolism and DNA and RNA synthesis. We also found genes related to UVA radiation resistance, to ion transport through membrane, and Mg transporters.

## Discussion

Sampling populations from across rainforest and savanna sites, our results show evidence of adaptation in *M. flexuosa* in several SNP loci. The OutFLANK approach identified 45 outlier loci with high *F*_*ST*_ values. Correlations of allele frequencies and climatic and soil variables indicated 45 loci putatively affecting adaptation. The test of recent selective sweeps provided evidence of more than 28 loci under natural selection, indicating evidence for selection as a driver of the evolutionary success of *M. flexuosa*, but did not support local adaptation.

Despite the climatic and soil differences between Amazonia, the Llanos, and the Cerrado, we found only slight differences in allele frequency at SNPs with selection footprints based on Bayenv 2 analysis (Figure 3), and most populations showed fixation of the same allele with selection signal (Figure 4, Supplementary Figures S12, 13 in Supplementary Appendix 2). The small shifts in allele frequency distributions among contrasting environments for most loci suggest selective sweeps of a single allele favored across most populations. We interpret these results as evidence for adaptation to the relatively stable microhabitat of *M. flexuosa*. Although the species occurs across highly variable macroclimatic conditions, from humid Amazonia rainforest to seasonally dry Cerrado savanna, the species is strictly associated to watercourses and swamps, named “veredas” in Cerrado, “moriches” in the Llanos in Colombia and Venezuela or “aguajales” in the Amazonian areas in Peru. In fact, the species is dependent on water and has physiological and morphological adaptations to germinate in swampy environments (Silva et al., 2014). Taken together, we suggest that the lack of or small differentiation in allele frequencies among populations from different ecosystems is due to the high specialization of *M. flexuosa* to swampy microhabitats, where selective sweeps maintain the same favorable alleles, despite the strong macroclimatic differences.

Bayesian clustering (Figure 1) showed differentiation among populations from different ecosystems in putatively neutral SNPs, but with some admixture between Amazonia and Cerrado, especially in populations from Araguaia/Tocantins, North Atlantic, and Caribbean river basins. In addition, AMOVA evidenced significant, but low differentiation among populations from different ecosystems, and isolation-by-distance is an important driver of genetic differentiation. Differentiation caused by genetic drift not only supports our hypothesis of homogenization among ecosystems due to selective sweeps, but also raises the hypothesis of migration spreading favorable alleles among populations leading to allele fixation (Yeaman and Whitlock, 2011; Savolainen et al., 2013).

Resilience to climate change can ensure evolutionary success (e.g., Whitlock and Barton, 1997). Although highly specialized to swampy microhabitats, *M. flexuosa* is resilient in the face of climatic change ensuring its evolutionary capacity. Suitable habitats for *M. flexuosa* may have been available during the Quaternary glacial cycling, mainly in the Amazon basin, and likely served as sources of migrants to colonize more unstable areas in Central Brazil during interglacial phases (de Lima et al., 2014; Melo et al., 2018). The demographic dynamics may have spread favorable mutations throughout populations leading to allele sharing of adaptive loci among populations. Similar patterns in allele frequency at adaptive loci were found in other widespread Neotropical tree species (Collevatti et al., 2019).

Moreover, despite the macroclimatic differences among populations from different ecosystems, there is a lack of strong latitudinal cline variation in climatic variables in the Neotropics (Figure 2), which may preclude the detection of adaptive signals in many loci, even in widespread species such as *M. flexuosa*. In fact, we identified adaptive selection correlated with mean annual temperature and mean diurnal range in temperature. Despite variation among populations, these variables show no clear geographic trend (Figure 2). The lack of strong latitudinal clines may explain the contrasting results compared to temperate species, for which adaptive selection has been reported for traits with strong latitudinal clines, such as photoperiod response and temperature response–related traits (e.g., Parchman et al., 2012; Yeaman et al., 2014; Hornoy et al., 2015). Top soil organic carbon and salinity have small differences among populations, and although top soil silt fraction and bulk density show larger differences among populations, we identified a patchy distribution among ecosystems, which may further mask the detection of local adaptive selection in traits related to soil adaptation. Nevertheless, all loci with adaptive signal are correlated with these soil variables. In addition, because most adaptive traits have quantitative inheritance (Fisher, 1930; Wright, 1935), the response of *M. flexuosa* to environmental variation may be polygenic, and thereby selection may cause only small changes in allele frequencies, causing weak selection signature on a single locus (Pritchard and Di Rienzo, 2010; Le Corre and Kremer, 2012).

### Selective Sweeps and Adaptation to Environmental Stress

Genes potentially affected by SNPs with selection signal have fundamental roles in cells and are usually highly conserved during evolution. Our analysis was based on functional annotation; thus, the real gene function must be confirmed using experimental assay such as reverse transcriptase–polymerase chain reaction to confirm gene expression. For instance, some genes highlighted in our analysis code for transduction membrane proteins (Wang et al., 2003) or are members of the ATP-binding cassette (ABC) or enzymes with catalytic activity. Adenosine 5’ triphosphate (ATP) and NADPH play a central role in metabolism, diffusing throughout the cell to the sites where energy is used for catalytic activities (Bonora et al., 2012). We also found adaptive signal for the proliferating cell nuclear antigen–like complex, which plays important roles in DNA replication and repair, including translesion synthesis, error-free damage bypass, break-induced replication, mismatch repair, and chromatin assembly (Boehm et al., 2016).

We identified genes under adaptive selection related to plant growth and adaptation to stress. The endomembrane trafficking protein MONENSIN SENSITIVITY1 (MonI), which is an important requirement for protein trafficking in cells and plant growth in *Arabidopsis thaliana* (Cui et al., 2014), was significantly correlated to variation in mean temperature diurnal range (Figure 4). The Mg transmembrane transport MRS2-4–related gene, essential for Mg homeostasis and the ability to adapt to a wide range of environmental conditions in *A. thaliana* (Oda et al., 2016), was correlated to top soil bulk density (Figure 4). This correlation indicates that *M. flexuosa* is adapted to variable and potentially stressful environments, enabling it to persist in adverse conditions.

We also found genes related to drought stress, such as the Rab scort protein geranyltransferase component, which is involved in abscisic acid and auxin signaling in *A. thaliana* (Johnson et al., 2005) and was correlated to mean temperature diurnal range (Figure 4). Interestingly, we found correlations with bioclimatic variables related to temperature, but not with precipitation, most likely because water via swampy habitat preference is required for *M. flexuosa* establishment. Thus, temperature variation among populations may impose higher constraints on the success of *M. flexuosa*.

It is important to note that populations from different ecosystems displayed the same fixed allele for these loci (Figure 4), indicating the role of selective sweeps shaping adaptation and spatial patterns in genetic diversity in *M. flexuosa*. On the other hand, we found weak evidence of local adaptation in different ecosystems, as evidence by few loci with exclusive alleles in just one ecosystem (Supplementary Figures S11, 12 in Supplementary Appendix 2). However, these loci potentially affect genes related to primary metabolism and are likely not directly related to local adaptation to climatic or soil conditions. Instead, they are most likely in LD with genes under selection.

Considering the number of loci scrutinized (16,262) and the transcriptome size of *M. flexuosa* (46,600 genes, Sarah et al., 2017), the number of loci detected with positive selection signal was low. It is important to note that the probe set did not cover all genes in *M. flexuosa* genome; thus, the number of loci that can be potentially detected is much lower than that in the transcriptome. It should be noted that the available methods to detect adaptive selection are based on the assumption that positive selection on a mutation leads to hard selective sweeps and therefore have little power to detect other types of selection such as background and balancing selection (Nielsen, 2005; Przeworski et al., 2005; Messer and Petrov, 2013). Thus, it is possible that our methodology allowed detection of only a fraction of loci under selection.

### Low Neutral Differentiation Among Ecosystems

The putatively neutral SNPs showed low but significant genetic differentiation among populations across (*F_C__T_* = 0.063) and within ecosystems (*F*_*SC*_ = 0.064). In addition, the Bayesian clustering showed some admixture among populations, especially from Amazonia and Cerrado. These results contrast with the higher differentiation for microsatellite loci (*F*_*CT*_ = 0.095; *F*_*SC*_ = 0.137), most likely due to higher mutation rates at microsatellite loci, leading to higher heterozygosity and differentiation. While SNPs are biallelic and evolve under an infinite sites model, microsatellites are multiallelic loci and evolve under stepwise and two-phase mutation models (Valdes et al., 1993; Di Rienzo et al., 1994). However, our findings showed similar patterns of spatial distribution of genetic clusters based on SNPs and microsatellites (Melo et al., 2018), with low admixture among ecosystems, suggesting an important role for genetic drift in shaping the spatial distribution of genetic diversity in *M. flexuosa*. Further, microsatellites, chloroplast sequences, and putatively neutral SNPs recovered a demographic scenario of multiple refugia present during the last glacial maximum (LGM, 26–19 kya; de Lima et al., 2014; Melo et al., 2018), reinforcing the hypothesis of the role of Quaternary climate change influencing the distribution of genetic diversity in *M. flexuosa*. Given the high fragmentation across current landscapes, it is likely that the genetic structure of *M. flexuosa* between Amazonia and Cerrado will remain in the future because of low gene flow among populations.

We also found higher frequency of mating between unrelated individuals than expected at random (negative inbreeding coefficient). It is important to note that *M. flexuosa* is a dioecious species, which therefore requires outcrossing. However, the high frequency of heterozygotes may also be the outcome of genotyping errors, leading to an overestimation of heterozygous genotypes (Chen et al., 2017). We believe this is unlikely here because we used standard procedures for SNP filtering (see Silva-Junior et al., 2018) with a minimum depth of aligned reads of three (median 26). Coverage depth of 1–2*x* has been found sufficient for accurate allele calls (Sims et al., 2014).

## Conclusion

Using target sequence capture SNP genotyping to generate data for over 16,000 SNPs, we unravel the evolutionary history of a hyperdominant palm species. Our findings suggest that selection together with migration led to the spread of alleles with higher fitness among populations from different ecosystems and promoted the evolutionary success of the widespread, highly abundant Neotropical palm *M. flexuosa* in swampy microhabitats.

## Data Availability Statement

Data and additional supporting information may be found in the online version of this article as supporting information. DNA datasets are available in GeneBank database (access number: BioProject ID PRJNA588981).

## Author Contributions

RC and CB conceived the experiment. RC and CB funded the work. WM conducted the experiment. WM, LV, EN, and RC performed the analysis including assembling, annotation, and curated the data. RC wrote the first draft of the manuscript. All co-authors contributed to the final version of the manuscript.

## Conflict of Interest

The authors declare that the research was conducted in the absence of any commercial or financial relationships that could be construed as a potential conflict of interest.

## Footnotes

1 http://www.worldclim.org/bioclim

2 http://www.fao.org/docrep/018/aq361e/aq361e.pdf

3 https://phytozome.jgi.doe.gov/pz/portal.html

## Supplementary Material

Supplementary Appendix 1 **Supplementary Tables S1–S12** with sampling locations, and Bayesian clustering and genome scan results.

Supplementary Appendix 2 **Supplementary Figures S1–S13** with details on Bayesian clustering and selection analyses.

Supplementary Appendix 3 **Supplementary Tables S1–S7** with SNP density, nucleotide diversity, OutFLANK result, and gene ontology annotation.
