# Supplementary material for: Selective Sweeps Lead to Evolutionary Success in an Amazonian Hyperdominant Palm
Source: Front Genet. 2020 Dec 23;11:596662. doi: 10.3389/fgene.2020.596662 (PMC7786001; doi:10.3389/fgene.2020.596662)
Supplement: Supplementary Appendix 2 — Supplementary Figures S1–S13 with details on Bayesian clustering and selection analyses. [file Data_Sheet_2.docx]

**Selective sweeps lead to evolutionary success in an Amazonian hyperdominant palm**

Warita A. Melo, Lucas D Vieira, Evandro Novaes, Christine D. Bacon, Rosane G. Collevatti

**______________________________________________________________________**

**Additional File S1 - Tables**

**Table S1**. Sampling location for the 22 populations of *Mauritia flexuosa* used for genetic analyses. N, number of individuals sampled. Geographical coordinates are in decimal degrees.

| **Ecosystem** | **River Basin** | **Locality** | **Country** | **Code** | **N** | **Latitude** | **Longitude** |
| --- | --- | --- | --- | --- | --- | --- | --- |
| **Amazonia** | **Amazon** | Comodoro, Mato Grosso | Brazil | COM | 8 | -13.596731 | -59.803833 |
|  |  | Estação Biológica EL Zafire, Leticia | Colombia | EZC | 12 | 4.008000 | -69.904722 |
|  |  | Imacita, Amazonas | Peru | IMA | 18 | -5.058324 | -78.338026 |
|  |  | Itacoatiara, Amazonas | Brazil | ITA | 10 | -3.041667 | -58.254167 |
|  |  | Manacapuru, Amazonas | Brazil | MAN | 14 | -3.416389 | -60.108444 |
|  |  | Reserva Extrativista do Alto Juruá, Acre | Brazil | PO1 | 16 | -9.121602 | -72.558234 |
|  |  | Presidente Figueiredo, Amazonas | Brazil | PRF | 9 | -2.205000 | -60.066083 |
|  |  | Reserva Ducke, Amazonas | Brazil | RED | 10 | -3.136389 | -59.116747 |
|  | **Caribbean** | Parque Aripo Savanna, Sangre Grande | Trinidad | AS1 | 10 | 10.716737 | -61.320239 |
|  | **North Atlantic** | Parc Amazonien de Guyane, Maripasoula | French Guiana | RAP | 12 | 3.291725 | -53.275716 |
|  |  | Rio Oyapock, Camopi | French Guiana | ROY | 12 | 3.292201 | -52.711972 |
| **Llanos** | **Orinoco** | San Martín, Meta | Colombia | SM1 | 16 | 3.552608 | -73.595256 |
|  |  | Yopal, Casanare | Colombia | YOP | 12 | 5.236855 | -72.542373 |
| **Cerrado** | **Parana-Paraguay** | Aporé, Goiás | Brazil | APO | 12 | -18.981031 | -51.916961 |
|  |  | Campo Grande, Mato Grosso | Brazil | CGR | 12 | -20.873260 | -54.822384 |
|  |  | Diamantino, Mato Grosso | Brazil | DIA | 12 | -14.458108 | -56.266389 |
|  | **São Francisco** | Parque Nacional Caverna do Peruaçu, Minas Gerais | Brazil | PNP | 9 | -15.017778 | -44.338625 |
|  | **Araguaia-Tocantins** | Águas Emendadas, Distrito Federal | Brazil | AGE | 12 | -15.344147 | -47.416631 |
|  |  | Grande Belém, Pará | Brazil | GBE | 12 | -1.458398 | -48.480686 |
|  |  | Nova Xavantina, Mato Grosso | Brazil | NXA | 12 | -14.743233 | -52.327928 |
|  |  | Paraíso do Tocantins, Tocantins | Brazil | PAT | 12 | -10.176719 | -48.925156 |
|  |  | Porto Franco, Amazonas | Brazil | PFR | 12 | -6.383433 | -47.377944 |
| **Total** |  |  |  | - | 264 | - | - |

**Table S2.** Factorial analysis for climate bioclimatic variables from the WorldClim Global Climate Bioclim database ([www.worldclim.org/bioclim](http://www.worldclim.org/bioclim)) for the 22 populations of *Mauritia flexuosa.* Bio1, mean annual temperature; Bio2, mean diurnal Range (mean of monthly (max temp - min temp); Bio3, isothermality (mean diurnal range /temperature annual range); Bio4, temperature seasonality; Bio5, maximum temperature of warmest month; Bio6, minimum temperature of coldest month; Bio7, Temperature Annual Range; Bio8, mean temperature of wettest quarter; Bio9, mean temperature of driest quarter; Bio10, mean temperature of warmest quarter; Bio11, mean temperature of coldest quarter; Bio12, annual precipitation; Bio13, precipitation of the wettest month; Bio14, precipitation of driest month; Bio15, precipitation seasonality; Bio16, precipitation of the wettest quarter; Bio17, precipitation of the driest quarter. SS, standard deviation. In bold, variables with higher correlation.

| **Loadings** | **MR1** | **MR2** | **MR3** | **MR4** |
| --- | --- | --- | --- | --- |
| **Bio 1** | **0.991** | -0.129 |  |  |
| **Bio 2** |  |  |  | **0.995** |
| **Bio 3** | -0.302 | 0.533 |  | 0.392 |
| **Bio 4** |  | -0.821 | -0.177 | 0.363 |
| **Bio 5** | 0.813 |  | 0.186 | 0.532 |
| **Bio 6** | 0.753 | 0.282 | 0.17 | -0.572 |
| **Bio 7** |  | -0.322 |  | 0.944 |
| **Bio 8** | 0.857 | -0.299 | -0.242 |  |
| **Bio 9** | 0.913 | 0.208 | 0.195 | -0.192 |
| **Bio 10** | 0.922 | -0.373 |  |  |
| **Bio 11** | 0.931 | 0.217 | 0.151 | -0.12 |
| **Bio 12** |  | 0.338 | 0.928 |  |
| **Bio 13** |  | 0.127 | 0.966 |  |
| **Bio 14** |  | **0.924** | 0.136 | -0.242 |
| **Bio 15** |  | -0.884 |  |  |
| **Bio 16** |  |  | **0.979** |  |
| **Bio 17** |  | 0.902 | 0.209 | -0.123 |
| **Bio 18** | -0.369 | 0.28 | 0.309 | 0.178 |
| **Bio 19** | 0.246 | 0.171 | 0.538 | -0.111 |
| **SS loadings** | 5.813 | 4.18 | 3.426 | 2.972 |
| **Proportion Variation** | 0.306 | 0.22 | 0.18 | 0.156 |
| **Cumulative Variation** | 0.306 | 0.526 | 0.706 | **0.863** |

**Table S3.** Factorial analysis for subsoil (30 – 100 cm) data related to soil fertility from the Harmonized World Soil Database (version 1.2, FAO/IIASA/ISRIC/ISS-CAS/JRC 2009), for the 22 populations of *Mauritia flexuosa*. T_SAND, Topsoil Sand Fraction; T_SILT, Topsoil Silt Fraction; T_CLAY, Topsoil Clay Fraction; T_REF_BULK_DENSITY, Topsoil Reference Bulk Density; T_OC, Topsoil Organic Carbon; T_PH_H2O, Topsoil pH (H2O); T_CEC_CLAY, Topsoil CEC (clay); T_CEC_SOIL, Topsoil CEC (soil); T_BS, Topsoil Base Saturation; T_TEB, Topsoil TEB; T_CACO3, Topsoil Calcium Carbonate; T_CASO4, Topsoil Gypsum; T_ESP, Topsoil Sodicity (ESP); T_ECE, Topsoil Salinity (Elco); S_GRAVEL, Subsoil Gravel Content; S_SAND, Subsoil Sand Fraction; S_SILT, Subsoil Silt Fraction; S_CLAY, Subsoil Clay Fraction; S_REF_BULK_DENSITY, Subsoil Reference Bulk Density; S_OC, Subsoil Organic Carbon; S_PH_H2O, Subsoil pH (H2O); S_CEC_CLAY, Subsoil CEC (clay); S_CEC_SOIL, Subsoil CEC (soil); S_BS, Subsoil Base Saturation ; S_TEB, Subsoil TEB; S_CACO3, Subsoil Calcium Carbonate; S_CASO4, Subsoil Gypsum; S_ESP, Subsoil Sodicity (ESP); S_ECE, Subsoil Salinity (ECe). SS, standard deviation. In bold, variables with higher correlation.

| **Loadings** | **MR1** | **MR2** | **MR3** | **MR4** |
| --- | --- | --- | --- | --- |
| **T_GRAVEL** | -0.488 | -0.145 | 0.105 |  |
| **T_SAND** | -0.763 | 0.134 |  | 0.6 |
| **T_SILT** | **0.956** |  |  | -0.155 |
| **T_CLAY** | -0.196 | -0.103 |  | -0.834 |
| **T_REF_BULK_DENSITY** | -0.28 | 0.175 | -0.103 | **0.906** |
| **T_OC** |  | -0.123 | **0.935** | 0.144 |
| **T_PH_H2O** | -0.242 | 0.822 |  | 0.34 |
| **T_CEC_CLAY** | 0.926 |  |  | 0.254 |
| **T_CEC_SOIL** | 0.391 |  |  | 0.922 |
| **T_BS** | 0.274 | 0.759 | 0.173 |  |
| **T_TEB** | 0.513 | 0.286 | 0.732 |  |
| **T_CACO3** | -0.123 | 0.723 | -0.165 | 0.112 |
| **T_CASO4** | -0.132 | 0.798 | -0.169 | 0.125 |
| **T_ESP** | 0.265 | -0.603 | 0.209 | 0.138 |
| **T_ECE** |  | **0.826** |  |  |
| **S_GRAVEL** | -0.511 | -0.109 |  | -0.127 |
| **S_SAND** | -0.822 | 0.148 |  | 0.462 |
| **S_SILT** | 0.954 | -0.198 | 0.11 |  |
| **S_CLAY** | -0.168 |  |  | -0.886 |
| **S_REF_BULK_DENSITY** | -0.428 | 0.141 |  | 0.819 |
| **S_OC** |  |  | 0.888 | 0.126 |
| **S_PH_H2O** |  | 0.742 |  | 0.291 |
| **S_CEC_CLAY** | 0.772 |  | 0.621 |  |
| **S_CEC_SOIL** | 0.706 |  | 0.667 | -0.183 |
| **S_BS** | 0.607 | 0.517 | 0.255 | 0.163 |
| **S_TEB** | 0.812 | 0.152 | 0.456 | -0.133 |
| **S_CACO3** | 0.117 | 0.595 |  |  |
| **S_CASO4** | 0.258 | 0.749 |  | -0.252 |
| **S_ESP** |  | -0.108 | 0.162 | 0.423 |
| **S_ECE** | 0.228 | 0.711 | 0.112 | -0.204 |
| **SS loadings** | 7.678 | 6.027 | 4.474 | 4.252 |
| **Proportion Var** | 0.256 | 0.201 | 0.149 | 0.142 |
| **Cumulative Var** | 0.256 | 0.457 | 0.606 | **0.748** |

**Table S4.** Association ratio (Q) of the main partition of *Mauritia flexuosa* clusters in each of the six clusters inferred by the Bayesian analysis implemented in the faststructure. N: number of individuals per population. In bold, Q ≥ 0.100.

| **Ecosystem** | **River Basin** | **Code** | **1** | **2** | **3** | **4** | **5** | **6** | **N** |
| --- | --- | --- | --- | --- | --- | --- | --- | --- | --- |
| **Amazonia** | **Amazon** | COM | **0.700** | 0.161 | 0.137 | 0.000 | 0.000 | 0.000 | 8 |
|  |  | EZC | **0.998** | 0.000 | 0.001 | 0.000 | 0.000 | 0.000 | 12 |
|  |  | IMA | **0.853** | 0.000 | 0.000 | 0.144 | 0.001 | 0.000 | 18 |
|  |  | ITA | **0.986** | 0.013 | 0.000 | 0.000 | 0.000 | 0.000 | 10 |
|  |  | MAN | **0.894** | 0.017 | 0.002 | 0.011 | 0.073 | 0.000 | 14 |
|  |  | PO1 | **0.884** | 0.106 | 0.009 | 0.000 | 0.000 | 0.000 | 16 |
|  |  | PRF | **0.919** | 0.080 | 0.000 | 0.000 | 0.000 | 0.000 | 9 |
|  |  | RED | **0.978** | 0.021 | 0.000 | 0.000 | 0.000 | 0.000 | 10 |
|  | **Caribbean** | AS1 | 0.000 | **0.999** | 0.000 | 0.000 | 0.000 | 0.000 | 10 |
|  | **North Atlantic** | RAP | 0.000 | **0.999** | 0.000 | 0.000 | 0.000 | 0.000 | 12 |
|  |  | ROY | 0.000 | **0.999** | 0.000 | 0.000 | 0.000 | 0.000 | 12 |
| **Llanos** | **Orinoco** | SM1 | 0.000 | 0.000 | 0.000 | **0.937** | 0.062 | 0.000 | 16 |
|  |  | YOP | 0.000 | 0.000 | 0.000 | **0.999** | 0.000 | 0.000 | 12 |
| **Cerrado** | **Parana-Paraguay** | APO | 0.000 | 0.006 | **0.993** | 0.000 | 0.000 | 0.000 | 12 |
|  |  | CGR | 0.000 | 0.000 | **0.999** | 0.000 | 0.000 | 0.000 | 12 |
|  |  | DIA | 0.066 | 0.016 | **0.911** | 0.000 | 0.005 | 0.000 | 12 |
|  | **São Francisco** | PNP | 0.000 | 0.000 | **0.999** | 0.000 | 0.000 | 0.000 | 9 |
|  | **Araguaia-Tocantins** | AGE | 0.000 | 0.000 | **0.999** | 0.000 | 0.000 | 0.000 | 12 |
|  |  | GBE | 0.000 | **0.999** | 0.000 | 0.000 | 0.000 | 0.000 | 12 |
|  |  | NXA | 0.000 | **0.928** | 0.071 | 0.000 | 0.000 | 0.000 | 12 |
|  |  | PAT | 0.000 | **0.999** | 0.000 | 0.000 | 0.000 | 0.000 | 12 |
|  |  | PFR | **0.919** | 0.080 | 0.000 | 0.000 | 0.000 | 0.000 | 12 |

**Table S5.** Association ratio (Q) of the minor partition of *Mauritia flexuosa* clusters in each of the five clusters inferred by the Bayesian analysis implemented in the faststructure. N: number of individuals per population. In bold, Q ≥ 0.100.

| **Ecosystem** | **River Basin** | **Code** | **1** | **2** | **3** | **4** | **5** | **N** |
| --- | --- | --- | --- | --- | --- | --- | --- | --- |
| **Amazonia** | **Amazon** | COM | **0.7127** | **0.1469** | **0.1327** | 0.0000 | 0.0076 | 8 |
|  |  | EZC | **0.9981** | 0.0000 | 0.0019 | 0.0000 | 0.0000 | 12 |
|  |  | IMA | **0.8498** | 0.0013 | 0.0000 | 0.1468 | 0.0019 | 18 |
|  |  | ITA | **0.9783** | 0.0216 | 0.0000 | 0.0000 | 0.0000 | 10 |
|  |  | MAN | **0.8887** | 0.0233 | 0.0025 | 0.0118 | 0.0736 | 14 |
|  |  | PO1 | **0.7831** | **0.1948** | 0.0096 | 0.0125 | 0.0000 | 16 |
|  |  | PRF | **0.8969** | **0.1030** | 0.0000 | 0.0000 | 0.0000 | 9 |
|  |  | RED | **0.9665** | 0.0334 | 0.0000 | 0.0000 | 0.0000 | 10 |
|  | **Caribbean** | AS1 | 0.0170 | **0.9829** | 0.0000 | 0.0000 | 0.0000 | 10 |
|  | **North Atlantic** | RAP | 0.0000 | **0.9999** | 0.0000 | 0.0000 | 0.0000 | 12 |
|  |  | ROY | 0.0038 | **0.9961** | 0.0000 | 0.0000 | 0.0000 | 12 |
| **Llanos** | **Orinoco** | SM1 | 0.0000 | 0.0000 | 0.0000 | **0.9374** | 0.0625 | 16 |
|  |  | YOP | 0.0000 | 0.0000 | 0.0000 | **0.9999** | 0.0000 | 12 |
| **Cerrado** | **Parana-Paraguay** | APO | 0.0000 | 0.0074 | **0.9925** | 0.0000 | 0.0000 | 12 |
|  |  | CGR | 0.0000 | 0.0000 | **0.9999** | 0.0000 | 0.0000 | 12 |
|  |  | DIA | 0.0683 | 0.0150 | **0.9111** | 0.0000 | 0.0055 | 12 |
|  | **São Francisco** | PNP | 0.0000 | 0.0000 | **0.9999** | 0.0000 | 0.0000 | 9 |
|  | **Araguaia-Tocantins** | AGE | 0.0000 | 0.0032 | **0.9961** | 0.0000 | 0.0006 | 12 |
|  |  | GBE | 0.0160 | **0.9812** | 0.0000 | 0.0000 | 0.0027 | 12 |
|  |  | NXA | **0.2113** | **0.7178** | 0.0663 | 0.0000 | 0.0046 | 12 |
|  |  | PAT | 0.0085 | **0.9904** | 0.0000 | 0.0000 | 0.0010 | 12 |
|  |  | PFR | 0.0000 | **0.9943** | 0.0056 | 0.0000 | 0.0000 | 12 |

**Table S6.** Association rate (Q) of the secondary partitions of *Mauritia flexuosa* clusters in each of the five clusters inferred by the Bayesian analysis implemented in the faststructure. N: number of individuals per population. In bold, Q ≥ 0.100.

|  |  |  |  |  | **Clusters (secondary partitions 1)** | | | | |  | **Clusters (secondary partitions 2)** | | | | |
| --- | --- | --- | --- | --- | --- | --- | --- | --- | --- | --- | --- | --- | --- | --- | --- |
| **Ecosystem** | **River Basin** | **Code** | **N** |  | **1** | **2** | **3** | **4** | **5** |  | **1** | **2** | **3** | **4** | **5** |
| **Amazonia** | **Amazon** | COM | 8 |  | **0.773** | 0.067 | 0.088 | 0.069 | 0.004 |  | **0.416** | **0.142** | 0.084 | 0.313 | 0.045 |
|  |  | EZC | 12 |  | **0.840** | 0.001 | **0.157** | 0.001 | 0.001 |  | **0.634** | **0.242** | 0.060 | 0.005 | 0.059 |
|  |  | IMA | 18 |  | **0.785** | 0.001 | **0.212** | 0.001 | 0.001 |  | **0.550** | **0.238** | **0.138** | 0.006 | 0.069 |
|  |  | ITA | 10 |  | **0.949** | 0.001 | 0.050 | 0.001 | 0.000 |  | **0.808** | **0.138** | 0.026 | 0.003 | 0.025 |
|  |  | MAN | 14 |  | **0.779** | 0.007 | **0.145** | 0.006 | 0.064 |  | **0.661** | **0.124** | 0.080 | **0.100** | 0.034 |
|  |  | PO1 | 16 |  | **0.826** | 0.001 | **0.172** | 0.001 | 0.001 |  | **0.195** | **0.469** | 0.031 | 0.006 | **0.299** |
|  |  | PRF | 9 |  | **0.599** | 0.001 | **0.400** | 0.001 | 0.001 |  | **0.590** | **0.105** | **0.220** | 0.004 | 0.082 |
|  |  | RED | 10 |  | **0.724** | 0.001 | **0.274** | 0.001 | 0.001 |  | **0.739** | 0.095 | 0.088 | 0.003 | 0.074 |
|  | **Caribbean** | AS1 | 10 |  | **0.933** | 0.000 | 0.066 | 0.000 | 0.000 |  | **0.368** | **0.523** | 0.034 | 0.003 | 0.072 |
|  | **North Atlantic** | RAP | 12 |  | **0.905** | 0.000 | 0.094 | 0.000 | 0.000 |  | **0.216** | **0.658** | 0.071 | 0.003 | 0.051 |
|  |  | ROY | 12 |  | **0.963** | 0.000 | 0.036 | 0.000 | 0.000 |  | **0.305** | **0.589** | 0.040 | 0.004 | 0.062 |
| **Llanos** | **Orinoco** | SM1 | 16 |  | **0.951** | 0.003 | 0.042 | 0.003 | 0.001 |  | **0.065** | **0.811** | 0.019 | 0.013 | 0.093 |
|  |  | YOP | 12 |  | **0.986** | 0.001 | 0.011 | 0.001 | 0.001 |  | **0.417** | **0.527** | 0.030 | 0.006 | 0.020 |
| **Cerrado** | **Parana-Paraguay** | APO | 12 |  | 0.084 | 0.003 | **0.910** | 0.003 | 0.001 |  | 0.065 | 0.068 | **0.315** | 0.006 | **0.546** |
|  |  | CGR | 12 |  | **0.167** | 0.012 | **0.808** | 0.012 | 0.001 |  | **0.130** | 0.062 | **0.147** | 0.046 | **0.616** |
|  |  | DIA | 12 |  | **0.129** | 0.050 | **0.767** | 0.050 | 0.005 |  | **0.148** | 0.036 | **0.591** | **0.164** | 0.061 |
|  | **São Francisco** | PNP | 9 |  | 0.083 | 0.001 | **0.913** | 0.001 | 0.003 |  | 0.039 | 0.075 | **0.184** | 0.006 | **0.697** |
|  | **Araguaia-Tocantins** | AGE | 12 |  | **0.113** | 0.013 | **0.855** | 0.013 | 0.006 |  | **0.172** | 0.051 | **0.622** | 0.050 | **0.105** |
|  |  | GBE | 12 |  | **0.710** | 0.077 | **0.125** | 0.081 | 0.006 |  | **0.289** | **0.210** | 0.073 | **0.367** | 0.062 |
|  |  | NXA | 12 |  | **0.502** | 0.080 | **0.336** | 0.081 | 0.002 |  | **0.224** | **0.121** | **0.235** | **0.352** | 0.069 |
|  |  | PAT | 12 |  | **0.571** | 0.000 | **0.428** | 0.000 | 0.000 |  | **0.257** | **0.385** | **0.141** | 0.003 | **0.213** |
|  |  | PFR | 12 |  | **0.602** | 0.006 | **0.384** | 0.006 | 0.001 |  | **0.299** | **0.337** | **0.162** | 0.026 | **0.175** |

**Table S7.** Probability values and 95% confidence intervals for the 10 subsets of closest simulated data. For all demographical scenarios we used the logistic approach in approximate Bayesian computation (ABC). In bold, the best-supported scenarios in comparison. These results are considering the generation time of 40 years (t525 for the Last Glacial Maximum).

| **Model** | **N** | **Expansion** | **Stability** | **Multiple Refugia** |
| --- | --- | --- | --- | --- |
| **Direct** | 100 | 0.3100 [0.0233,0.5967] | **0.4100 [0.1052,0.7148]** | 0.2800 [0.0017,0.5583] |
|  | 200 | 0.3000 [0.0160,0.5840] | **0.4300 [0.1231,0.7369]** | 0.2700 [0.0000,0.5452] |
|  | 300 | 0.3200 [0.0309,0.6091] | **0.4067 [0.1022,0.7111]** | 0.2733 [0.0000,0.5496] |
|  | 400 | 0.3125 [0.0252,0.5998] | **0.4175 [0.1118,0.7232]** | 0.2700 [0.0000,0.5452] |
|  | 500 | 0.3080 [0.0219,0.5941] | **0.4220 [0.1159,0.7281]** | 0.2700 [0.0000,0.5452] |
|  | 600 | 0.3050 [0.0196,0.5904] | **0.4267 [0.1201,0.7332]** | 0.2683 [0.0000,0.5430] |
|  | 700 | 0.3014 [0.0170,0.5858] | **0.4343 [0.1271,0.7415]** | 0.2643 [0.0000,0.5376] |
|  | 800 | 0.2987 [0.0151,0.5824] | **0.4300 [0.1231,0.7369]** | 0.2712 [0.0000,0.5468] |
|  | 900 | 0.2900 [0.0088,0.5712] | **0.4356 [0.1282,0.7429]** | 0.2744 [0.0000,0.5510] |
|  | 1000 | 0.2920 [0.0102,0.5738] | **0.4350 [0.1277,0.7423]** | 0.2730 [0.0000,0.5491] |
| **Logistic approach** | 100 | 0.0001 [0.0000,1.0000] | 0.0000 [0.0000,0.0008] | **0.9999 [0.9991,1.0000]** |
|  | 200 | 0.0001 [0.0000,1.0000] | 0.0000 [0.0000,0.0006] | **0.9999 [0.9994,1.0000]** |
|  | 300 | 0.0001 [0.0000,1.0000] | 0.0000 [0.0000,0.0005] | **0.9999 [0.9995,1.0000]** |
|  | 400 | 0.0001 [0.0000,1.0000] | 0.0000 [0.0000,0.0004] | **0.9999 [0.9995,1.0000]** |
|  | 500 | 0.0001 [0.0000,1.0000] | 0.0000 [0.0000,0.0004] | **0.9999 [0.9996,1.0000]** |
|  | 600 | 0.0001 [0.0000,1.0000] | 0.0000 [0.0000,0.0003] | **0.9999 [0.9996,1.0000]** |
|  | 700 | 0.0001 [0.0000,1.0000] | 0.0000 [0.0000,0.0003] | **0.9999 [0.9996,1.0000]** |
|  | 800 | 0.0001 [0.0000,1.0000] | 0.0000 [0.0000,0.0003] | **0.9999 [0.9997,1.0000]** |
|  | 900 | 0.0001 [0.0000,1.0000] | 0.0000 [0.0000,0.0003] | **0.9999 [0.9997,1.0000]** |
|  | 1000 | 0.0001 [0.0000,1.0000] | 0.0000 [0.0000,0.0003] | **0.9999 [0.9997,1.0000]** |

**Table S8.** SNPs with potential for adaptive selection based on the analysis of 22 populations, using Outflank software. The scaffold and SNP position is based on probe position in *Elaeis guianensis* genome. Affected genes were obtained using BlastX in Phytozome (https://phytozome.jgi.doe.gov/pz/portal.html). q, false discovery rate.

| **Scaffold** | **SNP position** | ***F_ST_*** | ***q*** | **Affected gene** | **Annotation** | **Function** |
| --- | --- | --- | --- | --- | --- | --- |
| Elaeis_136_8_0 | 73 | 1.000 | 0.007 | GSMUA_Achr4G25460_001 | PTHR23355 | Ribonuclease |
| Elaeis_136_8_0 | 78 | 1.000 | 0.007 | GSMUA_Achr4G25460_001 | PTHR23355 | Ribonuclease |
| Elaeis_182_1_212 | 12 | 1.000 | 0.007 | - | - | - |
| Elaeis_182_1_212 | 22 | 1.000 | 0.007 | - | - | - |
| Elaeis_218_1_132 | 24 | 0.839 | 0.031 | GSMUA_Achr3G07990_001 | GO:0005634 | Membrane-bounded organelle of eukaryotic cells |
| Elaeis_231_3_0 | 16 | 1.000 | 0.007 | - | - | - |
| Elaeis_231_3_7 | 81 | 1.000 | 0.007 | - | - | - |
| Elaeis_237_0_576 | 61 | 1.000 | 0.007 | - | - | - |
| Elaeis_237_6_79 | 67 | 1.000 | 0.007 | Aco018541 | PF00996 | GDP dissociation inhibitor |
| Elaeis_252_5_295 | 39 | 1.000 | 0.007 | - | - | - |
| Elaeis_252_5_295 | 49 | 1.000 | 0.007 | - | - | - |
| Elaeis_252_5_295 | 79 | 1.000 | 0.007 | - | - | - |
| Elaeis_257_8_31 | 15 | 1.000 | 0.007 | - | - | - |
| Elaeis_293_1_58 | 111 | 1.000 | 0.007 | - | - | - |
| Elaeis_299_2_0 | 23 | 0.816 | 0.035 | - | - | - |
| Elaeis_332_1_84 | 8 | 1.000 | 0.007 | GSMUA_Achr6G28980_001 | GO:0003824 | Catalysis of a biochemical reaction at physiological temperatures |
| Elaeis_391_1_0 | 54 | 0.867 | 0.024 | - | - | - |
| Elaeis_490_2_0 | 72 | 1.000 | 0.007 | Aco005118 | GO:0005524 | Interacting selectively and non-covalently with ATP |
| Elaeis_609_1_195 | 98 | 1.000 | 0.007 | - | - | - |
| Elaeis_609_1_195 | 99 | 1.000 | 0.007 | - | - | - |
| Elaeis_609_1_195 | 102 | 1.000 | 0.007 | - | - | - |
| Elaeis_637_5_6 | 82 | 1.000 | 0.007 | - | - | - |
| Elaeis_637_5_6 | 88 | 1.000 | 0.007 | - | - | - |
| Elaeis_637_5_6 | 91 | 1.000 | 0.007 | - | - | - |
| Elaeis_863_3_106 | 48 | 0.778 | 0.048 | - | - | - |
| Elaeis_985_1_84 | 70 | 0.859 | 0.025 | Solyc02g062560.2 | GO:0003824 | Catalysis of a biochemical reaction at physiological temperatures |
| Phoenix_252_7_60 | 61 | 0.804 | 0.040 | - | - | - |
| Sabal_17_1_58 | 89 | 1.000 | 0.007 | - | - | - |
| Sabal_252_3_9 | 68 | 0.833 | 0.031 | Spipo4G0029400 | GO:0005634 | A membrane-bounded organelle of eukaryotic cells |
| Sabal_296_8_0 | 99 | 1.000 | 0.007 | - | - | - |
| Sabal_32_3_84 | 37 | 0.876 | 0.023 | GSMUA_Achr3G21580_001 | GO:0006415 | Release of a polypeptide chain from the ribosome |
| Sabal_378_7_232 | 107 | 1.000 | 0.007 | - | - | - |
| Sabal_378_7_232 | 109 | 1.000 | 0.007 | - | - | - |
| Sabal_378_7_58 | 29 | 1.000 | 0.007 | - | - | - |
| Sabal_378_7_58 | 47 | 1.000 | 0.007 | - | - | - |
| Sabal_378_7_58 | 64 | 1.000 | 0.007 | - | - | - |
| Sabal_378_7_58 | 73 | 1.000 | 0.007 | - | - | - |
| Sabal_378_7_58 | 80 | 0.841 | 0.031 | - | - | - |
| Sabal_378_7_58 | 85 | 1.000 | 0.007 | - | - | - |
| Sabal_378_7_58 | 113 | 1.000 | 0.007 | - | - | - |
| Sabal_581_3_3 | 102 | 1.000 | 0.007 | - | - | - |
| Sabal_762_0_0 | 17 | 1.000 | 0.007 | - | - | - |
| Sabal_856_4_0 | 91 | 1.000 | 0.007 | - | - | - |
| Sabal_856_4_0 | 94 | 1.000 | 0.007 | - | - | - |
| Sabal_982_0_0 | 22 | 1.000 | 0.007 | - | - | - |

**Table S9.** SNPs with potential for adaptive selection based on Bayes factor for bioclimatic variables (BF > 100 for at least one bioclimatic variable) and correlation (Spearman’s correlation, |*ρ*| > 0.15) using Bayenv2 software. Scaffold and SNP position were obtained based on *Elaeis guianensis* genome. BIO1, mean annual temperature; BIO2, mean diurnal range (mean of monthly (max temp - min temp); BIO14, precipitation of driest month; BIO16, precipitation of wettest quarter. Affected genes were obtained using BlastX in Phytozome (https://phytozome.jgi.doe.gov/pz/portal.html). In bold, the variables with BF > 100.

| **Scaffold** | **Position** | **BF BIO01** | ***ρ* BIO01** | **BF BIO 02** | ***ρ* BIO 02** | **BF BIO 14** | ***ρ* BIO 14** | **BF BIO 16** | ***ρ* BIO16** | **Affected gene** | **Annotation** | **Function** |
| --- | --- | --- | --- | --- | --- | --- | --- | --- | --- | --- | --- | --- |
| Elaeis_1035_0_61 | 25 | **173.000** | **0.311** | 0.049 | 0.037 | 0.010 | 0.088 | 0.010 | 0.220 | Aco008085 | PF01765 | Ribosome recycling factor |
| Elaeis_164_0_63 | 87 | 0.052 | 0.014 | **2160.000** | **0.235** | 0.010 | 0.040 | 0.010 | 0.280 | Aco010979 | PF03164 | Trafficking protein Mon1 |
| Elaeis_1815_1_139 | 33 | 0.052 | -0.094 | **128.000** | **0.250** | 0.011 | 0.091 | 0.010 | 0.114 | Aco001871 | GO:0030896 | Conserved heterotrimeric complex of PCNA-like proteins that is loaded onto DNA at sites of DNA damage. |
| Elaeis_1901_2_0 | 20 | **207.000** | **0.274** | 0.093 | 0.066 | 0.010 | -0.088 | 0.010 | -0.084 | GSMUA_Achr10G12460_001 | GO:0016705 | Catalysis of an oxidation-reduction (redox) reaction in which hydrogen or electrons are transferred from each of two donors, and molecular oxygen is reduced or incorporated into a donor. |
| Elaeis_237_4_49 | 59 | 0.049 | 0.003 | **592.000** | **0.277** | 0.010 | -0.069 | 0.010 | 0.231 | GSMUA_Achr6G03390_001 | PTHR11787:SF4 | Rab proteins of the geranyltransferase component |
| Elaeis_2459_0_0 | 51 | 0.092 | -0.142 | **141.000** | **0.241** | 0.010 | -0.134 | 0.010 | 0.232 | 404193 | K12849 | Pre-mRNA-splicing factor 38A |
| Elaeis_362_0_57 | 67 | **846.000** | **0.307** | 0.144 | -0.058 | 0.010 | 0.059 | 0.010 | 0.010 | GSMUA_Achr8G22280_001 | PF07281 | Insulin-induced protein (INSIG) |
| Elaeis_378_1_84 | 67 | 0.059 | -0.048 | **387.000** | **0.209** | 0.010 | -0.250 | 0.010 | 0.168 | Aqcoe1G484200 | GO:0033588 | A heterohexameric protein complex that is involved in modification of wobble nucleosides in trna. The complex can associate physically with hyper phosphorylated RNA polymerase II; it contains two discrete heterotrimeric sub complexes. |
| Elaeis_421_2_192 | 97 | **111.000** | **-0.265** | 0.223 | 0.134 | 0.010 | -0.025 | 0.010 | -0.172 | GSMUA_Achr4G15010_001 | PTHR15885 | Uncharacterized |
| Elaeis_609_9_0 | 81 | **159.000** | **0.278** | 0.068 | 0.069 | 0.010 | 0.063 | 0.010 | -0.110 | Aco011489 | GO:0008375 | Catalysis of the transfer of an N-acetylglucosaminyl residue from UDP-N-acetyl-glucosamine to a sugar. |
| Elaeis_630_1_0 | 45 | 0.064 | -0.097 | **404.000** | **0.231** | 0.010 | 0.044 | 0.010 | 0.155 | - | - | - |
| Elaeis_673_15_0 | 33 | **220.000** | **0.358** | 0.077 | 0.005 | 0.010 | 0.050 | 0.010 | 0.137 | GRMZM5G855347 | GO:0046872 | Interacting selectively and non-covalently with any metal ion. |
| Elaeis_758_10_0 | 65 | **119.000** | **-0.263** | 0.168 | 0.145 | 0.010 | -0.077 | 0.010 | -0.092 | GSMUA_Achr10G14520_001 | GO:0009058 | The chemical reactions and pathways resulting in the formation of substances; typically the energy-requiring part of metabolism in which simpler substances are transformed into more complex ones. |
| Elaeis_7_9_61 | 60 | 0.057 | 0.008 | **904.000** | **0.387** | 0.010 | -0.116 | 0.010 | 0.125 | Aco019921 | GO:0006508 | The hydrolysis of proteins into smaller polypeptides and/or amino acids by cleavage of their peptide bonds. |
| Elaeis_863_0_378 | 33 | 0.264 | 0.001 | **169.000** | **0.262** | 0.010 | 0.084 | 0.010 | -0.057 | GSMUA_Achr2G13920_001 | GO:0006508 | The hydrolysis of proteins into smaller polypeptides and/or amino acids by cleavage of their peptide bonds. |
| Elaeis_985_6_0 | 107 | **108.000** | **0.335** | 0.077 | 0.087 | 0.010 | 0.125 | 0.010 | 0.011 | Eucgr.I02404 | GO:0003824 | Catalysis of a biochemical reaction at physiological temperatures. In biologically catalyzed reactions, the reactants are known as substrates, and the catalysts are naturally |
| Phoenix_225_6_228 | 58 | **641.000** | **0.239** | 0.565 | 0.287 | 0.010 | -0.122 | 0.010 | -0.089 | Aco006501 | GO:0008270 | Interacting selectively and non-covalently with zinc (Zn) ions. |

**Table S10.** SNPs with potential for adaptive selection based on Bayes factor for soil variables (BF > 100 for at least one soil variable) and correlation (Spearman’s correlation, |*ρ*| > 0.15) using Bayenv2 software. Scaffold and SNP position were obtained based on *Elaeis guineensis* genome. T_REF_BULK_DENSITY, Topsoil Reference Bulk Density; T_ECE, Topsoil Salinity; T_OC, Topsoil Organic Carbon; T_SILT, Topsoil Silt Fraction. Affected genes were obtained using BlastX in Phytozome (https://phytozome.jgi.doe.gov/pz/portal.html). In bold, the variables with BF > 100.

|  |  | **T_REF_BULK_DENSITY** | | | **T_ECE** | | | **T_OC** | | | | **T_SILT** | | | | |  | | |  | |  | | |  |  |
| --- | --- | --- | --- | --- | --- | --- | --- | --- | --- | --- | --- | --- | --- | --- | --- | --- | --- | --- | --- | --- | --- | --- | --- | --- | --- | --- |
| **Scaffold** | **Position** | | **BF** | ***ρ*** | | **BF** | ***ρ*** | | **BF** | | ***ρ*** | | **BF** | | ***ρ*** | | | **Affected gene** | | | **Annotation** | | **Function** | | |  |
| Elaeis_136_24_0 | 45 | | **2030** | **-0.371** | | 1.37 | -0.358 | | 0.0451 | | 0.0686 | | 0.892 | | 0.139 | | | Aco011861 | | | PTHR23355 | | Ribonuclease | | |  |
| Elaeis_139_3_132 | 89 | | **487** | **-0.469** | | 1.98 | -0.387 | | 0.122 | | 0.0675 | | 0.00992 | | 0.139 | | | GSMUA_Achr1G20540_001 | | | PF05705 | | Eukaryotic protein of unknown function (DUF829) | | |  |
| Elaeis_1484_2_60 | 69 | | **109** | **-0.364** | | 1.47 | -0.276 | | 0.0276 | | -0.0341 | | 0.0101 | | 0.0949 | | | PGSC0003DMG400010521 | | | PF10184 | | Uncharacterized conserved protein (DUF2358) | | |  |
| Elaeis_1877_0_268 | 31 | | **116** | **-0.281** | | 1.09 | -0.219 | | 0.0575 | | 0.0171 | | 0.0099 | | 0.0177 | | | GSMUA_Achr9G18630_001 | | | KOG2896 | | UV radiation resistance associated protein | | |  |
| Elaeis_226_7_57 | 72 | | **111** | **-0.352** | | 1.27 | -0.297 | | 0.0276 | | 0.078 | | 0.0117 | | 0.182 | | | Aco000617 | | | PF12874 | | Zinc-finger of C2H2 type | | |  |
| Elaeis_24_0_305 | 81 | | 0.588 | -0.0157 | | 1.09 | -0.0407 | | 0.0647 | | 0.276 | | **303** | | **0.115** | | | GSMUA_Achr1G13560_001 | | | GO:0005576 | | The space external to the outermost structure of a cell. For cells without external protective or external encapsulating structures this refers to space outside of the plasma membrane. This term covers the host cell environment outside an intracellular parasite. | | |  |
| Elaeis_281_3_569 | 50 | | **155** | **-0.331** | | 1.18 | -0.236 | | 0.0249 | | 0.0703 | | 0.00993 | | 0.116 | | |  | | | - | | - | | |  |
| Elaeis_340_2_66 | 73 | | **199** | **-0.438** | | 1.29 | -0.348 | | 0.074 | | 0.202 | | 0.0109 | | 0.257 | | | GSMUA_Achr4G29320_001 | | | PTHR13890:SF2 | | Magnesium transporter MRS2-4-related | | |  |
| Elaeis_340_3_165 | 28 | | **131** | **-0.27** | | 1.19 | -0.297 | | 0.0247 | | 0.189 | | 0.0101 | | 0.153 | | | evm_27.TU.AmTr_v1.0_scaffold00056.31 | | | PTHR13890:SF2 | | Magnesium transporter MRS2-4-related | | |  |
| Elaeis_673_13_55 | 49 | | **101** | **-0.389** | | 1.49 | -0.344 | | 0.0824 | | 0.0899 | | 0.0099 | | 0.0642 | | | Aco003959 | | | GO:0030001 | | The directed movement of metal ions, any metal ion with an electric charge, into, out of or within a cell, or between cells, by means of some agent such as a transporter or pore. | | |  |
| Elaeis_740_5_0 | 33 | | **133** | **-0.162** | | 1.02 | -0.102 | | 0.0308 | | -0.0343 | | 0.00991 | | 0.059 | | | GSMUA_Achr11G19340_001 | | | PF12697 | | Alpha/beta hydrolase family | | |  |
| Elaeis_758_3_31 | 59 | | **106** | **-0.382** | | 1.45 | -0.242 | | 0.183 | | -0.0354 | | 0.00991 | | -0.0375 | | | Aco010714 | | | GO:0005524 | | Interacting selectively and non-covalently with ATP, adenosine 5'-triphosphate, a universally important coenzyme and enzyme regulator. | | |  |
| Elaeis_7_9_0 | 90 | | **477** | **-0.445** | | 1.75 | -0.34 | | 0.0346 | | -0.0104 | | 0.00992 | | 0.116 | | | evm_27.TU.AmTr_v1.0_scaffold00041.27 | | | GO:0006508 | | The hydrolysis of proteins into smaller polypeptides and/or amino acids by cleavage of their peptide bonds. | | |  |
| Elaeis_7_9_61 | 29 | | **145** | **-0.394** | | 1.7 | -0.327 | | 0.0524 | | -0.0257 | | 0.0099 | | 0.153 | | | Aco019921 | | | GO:0006508 | | The hydrolysis of proteins into smaller polypeptides and/or amino acids by cleavage of their peptide bonds. | | |  |
| Elaeis_808_7_39 | 88 | | **759** | **-0.262** | | 1.12 | -0.226 | | 0.0474 | | 0.0865 | | 0.0099 | | 0.0843 | | | GSMUA_Achr3G24420_001 | | | PF14901 | | Cleavage inducing molecular chaperone | | |  |
| Elaeis_897_10_0 | 102 | | **373** | **-0.391** | | 1.82 | -0.333 | | 0.0389 | | 0.0149 | | 0.00992 | | -0.0668 | | | AL48U10090 | | | GO:0006470 | | The process of removing one or more phosphoric residues from a protein. | | |  |
| Elaeis_897_10_52 | 50 | | **139** | **-0.393** | | 1.94 | -0.356 | | 0.0306 | | -0.00958 | | 0.0099 | | -0.0701 | | | GSMUA_Achr2G16570_001 | | | SSF90257 | | Myosin rod fragments | | |  |
| Elaeis_948_3_60 | 60 | | **246** | **-0.464** | | 1.68 | -0.346 | | 0.056 | | 0.0591 | | 0.0151 | | 0.27 | | | GSMUA_Achr11G18240_001 | | | GO:0005975 | | The chemical reactions and pathways involving carbohydrates, any of a group of organic compounds based of the general formula Cx(H2O)y. Includes the formation of carbohydrate derivatives by the addition of a carbohydrate residue to another molecule. | | | |
| Elaeis_958_9_65 | 96 | | **242** | **-0.434** | | 1.76 | -0.312 | | 0.0729 | | -0.0109 | | 0.00993 | | 0.0651 | | | Cucsa.160210 | | | PF10250 | | GDP-fucose protein O-fucosyltransferase | | | |
| Elaeis_989_5_38 | 113 | | **424** | **-0.43** | | 2.08 | -0.342 | | 0.0371 | | -0.0218 | | 0.0099 | | | 0.0763 | | Pavir.J02474 | | | GO:0016817 | | Catalysis of the hydrolysis of any acid anhydride. | | | |
| Sabal_148_4_102 | 50 | | **616** | **-0.44** | | 2.15 | -0.358 | | 0.0632 | | 0.00248 | | 0.0099 | | | -0.144 | | GSMUA_Achr10G14610_001 | | | PTHR22835 | | Zinc finger FYVE domain containing protein | | | |
| Sabal_148_4_51 | 101 | | **4090** | **-0.452** | | 2.33 | -0.38 | | 0.0449 | | 0.0276 | | 0.0101 | | | 0.0614 | | GSMUA_Achr10G14610_001 | | | PTHR22835 | | Zinc finger FYVE domain containing protein | | | |
| Sabal_334_4_0 | 24 | | **165** | **-0.367** | | 1.11 | -0.209 | | 0.035 | | -0.0588 | | 0.00991 | | | 0.0442 | | - | | | - | | - | | | |
| Sabal_736_6_229 | 30 | | **4380** | **-0.466** | | 2.23 | -0.379 | | 0.0542 | 0.0297 | | | | 0.0099 | | 0.0284 | | | Aqcoe5G407200 | | GO:0005751 | | | A protein complex located in the mitochondrial inner membrane that forms part of the mitochondrial respiratory chain. Contains the 13 polypeptide subunits of cytochrome c oxidase, including cytochrome a and cytochrome a3. Catalyzes the oxidation of reduced cytochrome c by dioxygen (O2). | | |

**Table S11**. Environmental variables used in Bayenv2, for each sampled population. Bio4, temperature seasonality; Bio5, maximum temperature of warmest month; Bio1, mean annual temperature; Bio2, mean diurnal range (mean of monthly (max temp - min temp); Bio14, precipitation of driest month; Bio16, precipitation of wettest quarter; T_ECE, Topsoil Salinity (Elco); T_REF_BULK_DENSITY, Topsoil Reference Bulk Density; T_OC, Topsoil Organic Carbon; T_SILT, Topsoil Silt Fraction. SD, standard variation.

| **Ecosystem** | **River Basin** | **Code** | **Latitude** | **Longitude** | **Bio 1** | **Bio 2** | **Bio 14** | **Bio16** | **T_ECE** | **S_REF_BULK_DENSITY** | **T_OC** | **T_SILT** |
| --- | --- | --- | --- | --- | --- | --- | --- | --- | --- | --- | --- | --- |
| **Amazonia** | **Amazon** | **COM** | -13.597 | -59.804 | 23.674 | 10.658 | 637.108 | 1.724 | 0.004 | 1.404 | 1.382 | 9.789 |
|  |  | **EZC** | 4.008 | -69.905 | 24.384 | 7.880 | 686.450 | 26.379 | - | 1.280 | 1.019 | 41.585 |
|  |  | **IMA** | -5.058 | -78.338 | 20.770 | 9.552 | 490.255 | 22.298 | 0.075 | 1.323 | 2.205 | 30.850 |
|  |  | **ITA** | -3.042 | -58.254 | 24.757 | 7.942 | 892.949 | 28.752 | - | 1.257 | 0.987 | 36.138 |
|  |  | **MAN** | -3.416 | -60.108 | 24.773 | 7.790 | 955.103 | 29.973 | - | 1.306 | 1.140 | 46.193 |
|  |  | **PO1** | -9.122 | -72.558 | 24.398 | 10.650 | 512.415 | 14.412 | 0.001 | 1.244 | 1.128 | 45.378 |
|  |  | **PRF** | -2.205 | -60.066 | 24.653 | 7.584 | 943.093 | 30.713 | - | 1.344 | 1.078 | 11.800 |
|  |  | **RED** | -3.136 | -59.117 | 24.765 | 7.896 | 940.023 | 28.371 | - | 1.296 | 1.066 | 44.478 |
|  | **Caribbean** | **AS1** | 10.717 | -61.320 | 25.427 | 0.686 | 301.885 | 8.701 | - | 1.189 | 10.872 | 36.479 |
|  | **North Atlantic** | **RAP** | 3.292 | -53.276 | 23.596 | 7.466 | 462.016 | 36.344 | - | 1.274 | 0.976 | 8.526 |
|  |  | **ROY** | 3.292 | -52.712 | 23.496 | 7.126 | 475.344 | 38.204 | - | 1.273 | 1.000 | 8.133 |
| **Llanos** | **Orinoco** | **SM1** | 3.553 | -73.595 | 24.253 | 7.895 | 761.495 | 35.020 | - | 1.318 | 1.602 | 33.570 |
|  |  | **YOP** | 5.237 | -72.542 | 18.212 | 7.121 | 1,236.233 | 21.561 | - | 1.368 | 13.508 | 25.561 |
| **Cerrado** | **Parana-Paraguay** | **APO** | -18.981 | -51.917 | 22.606 | 9.780 | 740.397 | 2.465 | 0.032 | 1.414 | 0.884 | 7.730 |
|  |  | **CGR** | -20.873 | -54.822 | 23.425 | 10.491 | 718.981 | 5.755 | 0.035 | 1.375 | 0.884 | 12.426 |
|  |  | **DIA** | -14.458 | -56.266 | 24.950 | 9.779 | 691.124 | 0.836 | 0.012 | 1.378 | 1.336 | 11.189 |
|  | **São Francisco** | **PNP** | -15.018 | -44.339 | 23.329 | 8.253 | 354.631 | 9.592 | 0.024 | 1.386 | 0.928 | 14.298 |
|  | **Araguaia-Tocantins** | **AGE** | -15.344 | -47.417 | 22.035 | 8.306 | 607.819 | 3.784 | - | 1.293 | 1.224 | 14.954 |
|  |  | **GBE** | -1.458 | -48.481 | 25.052 | 6.149 | 1,078.074 | 27.662 | - | 1.397 | 0.975 | 19.431 |
|  |  | **NXA** | -14.743 | -52.328 | 24.754 | 9.195 | 728.090 | 0.585 | - | 1.504 | 0.841 | 10.390 |
|  |  | **PAT** | -10.177 | -48.925 | 25.355 | 8.712 | 1,020.415 | 0.629 | - | 1.363 | 0.785 | 12.172 |
|  |  | **PFR** | -6.383 | -47.378 | 25.193 | 7.388 | 1,398.909 | 4.074 | 0.016 | 1.433 | 0.794 | 12.842 |
| **Mean** |  |  |  |  | 23.812 | 8.105 | 756.037 | 17.174 | 0.009 | 1.337 | 2.119 | 22.451 |
| **SD** |  |  |  |  | 1.700 | 2.073 | 280.722 | 13.565 | 0.018 | 0.073 | 3.300 | 14.008 |

**Table S12** Evidence for selective sweeps based on composite likelihood using the parametric test implemented in SweepFinder2 for the 22 populations of *Mauritia flexuosa*. The values are the location and the calculated composite likelihood. Only the most extreme signals above the significance cut-off are summarized in terms of the most probable location of the sweep to the nearest gene locus annotated. Distance is the minimum distance of the location of the sweeps in the populations to the closest locus. 0-values mean that the location of the most extreme signal is within the gene locus. Affected genes were obtained using BlastX in Phytozome (https://phytozome.jgi.doe.gov/pz/portal.html).

| **Affected Loci** | **AGE** | **APO** | **AS1** | **CGR** | **COM** | **DIA** | **EZC** | **GBE** | **IMA** | **ITA** | **MAN** | **NXA** | **PAT** | **PFR** | **PNP** | **PO** | **PRF** | **RAP** | **RED** | **ROY** | **SM1** | **YOP** | **Affected gene** | **Annotation** | **Function** |
| --- | --- | --- | --- | --- | --- | --- | --- | --- | --- | --- | --- | --- | --- | --- | --- | --- | --- | --- | --- | --- | --- | --- | --- | --- | --- |
| **Elaeis_237_4_49** | 107 (1.750) | 90 (1.862) | 107 (1.659) | 107 (1.439) | 107 (1.766) | 107 (2.944) | 107 (1.956) | 107 (1.482) | 107 (2.477) | 106 (2.385) | 105 (3.097) | 107 (3.579) | 107 (1.586) | 90 (1.773) | 90 (1.830) | 91 (1.329) | 53 (2.053) | 107 (1.416) | 107 (1.754) | 107 (1.414) | 91 (1.287) | 107 (1.472) | Gsmua_achr6g03390_001 | PTHR11787:SF4 | Rab proteins geranylgeranyltransferase component a |
| **Elaeis_2459_0_0** | - | 47 (1.251) | - | - | 59 (1.641) | - | 107 (1.266) | - | 44 (1.401) | 44 (1.672) | 102 (1.459) | 47 (1.287) | - | - | - | - | - | - | - | - | - | 97 (1.484) | 404193 | K12849 | Pre-mRNA-splicing factor 38A |
| **Elaeis_362_0_57** | - | - | - | - | 39 (1.425) | - | - | - | - | - | - | - | - | - | - | - | - | - | - | - | 108 (1.242) | - | Gsmua_achr8g22280_001 | PF07281 | Insulin-induced protein (INSIG) |
| **Elaeis_378_1_84** | - | - | - | - | - | - | - | 66 (1.984) | - | - | - | 66 (1.331) | - | - | - | - | - | - | 67 (1.220) | - | - | - | Aqcoe1g484200 | GO:0033588 | A heterohexameric protein complex that is involved in modification of wobble nucleosides in trna. The complex can associate physically with hyperphosphorylated RNA polymerase II; it contains two discrete heterotrimeric subcomplexes. |
| **Elaeis_630_1_0** | - | - | - | 112 (1.159) | - | - | - | - | - | - | - | - | - | - | - | - | - | - | - | - | 14 (1.214) | - | - | - | - |
| **Elaeis_673_15_0** | - | - | - | - | - | - | - | - | - | - | - | - | - | - | - | - | - | - | - | - | 33 (2.111) | - | Grmzm5g855347 | GO:0046872 | Interacting selectively and non-covalently with any metal ion. |
| **Elaeis_758_10_0** | - | - | - | - | - | - | - | - | - | - | - | - | 18 (1.387) | - | - | - | - | - | - | - | 23 (1.343) | - | Gsmua_achr10g14520_001 | GO:0009058 | The chemical reactions and pathways resulting in the formation of substances; typically the energy-requiring part of metabolism in which simpler substances are transformed into more complex ones. |
| **Elaeis_985_6_0** | - | - | - | - | - | - | - | - | - | - | - | - | - | - | - | - | - | - | - | - | 107 (1.765) | - | Eucgr.i02404 | GO:0003824 | Catalysis of a biochemical reaction at physiological temperatures. In biologically catalyzed reactions, the reactants are known as substrates, and the catalysts are naturally |
| **Phoenix_225_6_228** | - | - | - | - | 35 (1.483) | 35 (1.260) | - | - | - | - | - | - | - | - | - | - | - | - | - | - | - | - | Aco006501 | GO:0008270 | Interacting selectively and non-covalently with zinc (Zn) ions. |
| **Elaeis_136_24_0** | - | - | - | - | 45 (1.441) | - | - | - | - | - | - | - | - | - | - | - | - | - | - | 72 (1.136) | - | 45 (1.202) | Aco011861 | PTHR23355 | Ribonuclease |
| **Elaeis_139_3_132** | 25 (1.699) | 15 (1.416) | 89 (1.134) | - | 25 (2.241) | 14 (1.344) | 44 (2.214) | 25 (1.176) | 44 (1.628) | - | 47 (1.125) | - | - | - | 9 (1.267) | 25 (1.349) | 89 (1.296) | 25 (1.379) | 9 (1.290) | 89 (1.271) | - | - | Gsmua_achr1g20540_001 | PF05705 | Eukaryotic protein of unknown function (duf829) |
| **Elaeis_1484_2_60** | - | - | - | - | - | 57(1.480) | - | - | 30 (1.258) | - | - | - | - | 69 (1.194) | - | - | - | - | - | - | 69 (2.081) | - | Pgsc0003dmg400010521 | PF10184 | Uncharacterized conserved protein (duf2358) |
| **Elaeis_1877_0_268** | 31 (1.849) | 31 (1.596) | - | - | 31 (2.371) | 31 (1.363) | - | - | 59 (1.221) | 31 (1.253) | 7 (1.271) | - | - | - | - | - | 7 (1.332) | - | 7 (1.477) | - | - | - | Gsmua_achr9g18630_001 | KOG2896 | UV radiation resistance associated protein |
| **Elaeis_226_7_57** | - | - | - | - | - | - | - | - | 19 (1.712) | - | - | - | - | - | - | - | - | - | - | - | - | - | Aco000617 | PF12874 | Zinc-finger of c2h2 type |
| **Elaeis_281_3_569** | - | - | - | - | - | - | - | - | - | - | - | - | - | - | - | - | - | - | - | - | 50 (1.129) | - | - | - | - |
| **Elaeis_340_2_66** | - | - | - | - | - | - | - | - | - | - | - | - | - | - | - | - | - | - | - | - | 73 (1.841) | - | Gsmua_achr4g29320_001 | PTHR13890:SF2 | Magnesium transporter mrs2-4-related |
| **Elaeis_340_3_165** | 28 (1.889) | 22 (1.868) | - | - | 16 (2.084) | 53 (1.312) | 14 (3.262) | - | 16 (1.280) | 16 (1.386) | 51 (1.397) | 37 (1.455) | 16 (1.421) | - | 16 (1.730) | 15 (1.817) | 46 (1.560) | - | 16 (1.507) | - | 53 (1.450) | 73 (1.226) | Evm_27.tu.amtr_v1.0_scaffold00056.31 | PTHR13890:SF2 | Magnesium transporter mrs2-4-related |
| **Elaeis_673_13_55** | - | - | - | - | - | - | - | - | - | - | - | - | - | - | - | - | - | - | - | - | 66 (1.193) | - | Aco003959 | GO:0030001 | The directed movement of metal ions, any metal ion with an electric charge, into, out of or within a cell, or between cells, by means of some agent such as a transporter or pore. |
| **Elaeis_740_5_0** | - | - | - | - | - | - | - | - | - | - | - | - | - | - | - | - | - | - | - | - | 56 (1.125) | - | Gsmua_achr11g19340_001 | PF12697 | Alpha/Beta hydrolase family |
| **Elaeis_758_3_31** | 24 (1.436) | - | - | - | 24 (1.826) | - | - | 23 (1.135) | - | - | 23 (1.485) | - | - | - | - | - | - | - | - | - | - | - | Aco010714 | GO:0005524 | Interacting selectively and non-covalently with ATP, adenosine 5'-triphosphate, a universally important coenzyme and enzyme regulator. |
| **Elaeis_7_9_0** | - | - | - | - | - | - | - | 21 (1.146) | - | - | - | - | - | - | - | - | - | - | - | - | - | - | Evm_27.tu.amtr_v1.0_scaffold00041.27 | GO:0006508 | The hydrolysis of proteins into smaller polypeptides and/or amino acids by cleavage of their peptide bonds. |
| **Elaeis_808_7_39** | 21 (1.293) | - | - | 21 (1.359) | - | - | - | - | - | - | - | - | - | 21 (1.160) | - | - | - | - | - | - | 88 (2.429) | - | Gsmua_achr3g24420_001 | PF14901 | Cleavage inducing molecular chaperone |
| **Elaeis_897_10_0** | - | - | - | - | - | - | - | - | - | - | - | - | - | - | - | - | - | - | - | - | 102 (2.015) | - | Al48u10090 | GO:0006470 | The process of removing one or more phosphoric residues from a protein. |
| **Elaeis_897_10_52** | - | - | - | 29 (1.142) | - | - | - | - | - | - | - | - | - | - | - | - | - | - | - | - | 38 (1.443) | - | Gsmua_achr2g16570_001 | SSF90257 | Myosin rod fragments |
| **Elaeis_989_5_38** | - | - | - | - | - | - | - | - | - | - | - | - | 115 (1.586) | - | - | - | - | - | - | - | - | - | Pavir.j02474 | GO:0016817 | Catalysis of the hydrolysis of any acid anhydride. |
| **Sabal_148_4_51** | - | - | - | - | - | - | - | - | - | - | - | - | - | - | - | - | - | - | - | - | 96 (2.051) | - | Gsmua_achr10g14610_001 | PTHR22835 | Zinc finger fyve domain containing protein |
| **Sabal_334_4_0** | - | 101 (1.691) | - | 110 (1.307) | - | - | - | - | - | - | - | - | - | - | - | - | - | - | - | - | 76 (1.194) | - | - | - | - |
| **Sabal_736_6_229** | 18 (4.380) | 15 (4.394) | 15 (3.145) | 31 (2.286) | 15 (4.784) | 19 (3.030) | 24 (3.445) | 15 (2.159) | 15 (2.619) | 12 (1.956) | 15 (2.418) | 15 (5.245) | 12 (1.742) | 18 (3.302) | 33 (2.156) | 13 (2.582) | 12 (1.927) | 12 (2.063) | 13 (2.792) | 13 (2.865) | 14 (1.739) | 18 (1.700) | Aqcoe5g407200 | GO:0005751 | A protein complex located in the mitochondrial inner membrane that forms part of the mitochondrial respiratory chain. Contains the 13 polypeptide subunits of cytochrome c oxidase, including cytochrome a and cytochrome a3. Catalyzes the oxidation of reduced cytochrome c by dioxygen (O2). |
| **Number of loci** | **7** | **7** | **3** | **6** | **10** | **7** | **5** | **6** | **8** | **5** | **7** | **5** | **5** | **4** | **4** | **4** | **5** | **3** | **6** | **4** | **18** | **5** | **-** | **-** | **134** |
