# Supplementary material for: Selective Sweeps Lead to Evolutionary Success in an Amazonian Hyperdominant Palm
Source: Front Genet. 2020 Dec 23;11:596662. doi: 10.3389/fgene.2020.596662 (PMC7786001; doi:10.3389/fgene.2020.596662)
Supplement: Supplementary Appendix 3 — Supplementary Tables S1–S7 with SNP density, nucleotide diversity, OutFLANK result, and gene ontology annotation. [file Data_Sheet_3.docx]

**Selective sweeps lead to evolutionary success in an Amazonian hyperdominant palm**

Warita A. Melo, Lucas D Vieira, Evandro Novaes, Christine D. Bacon, Rosane G. Collevatti

**Additional File S2 - Figures**

**
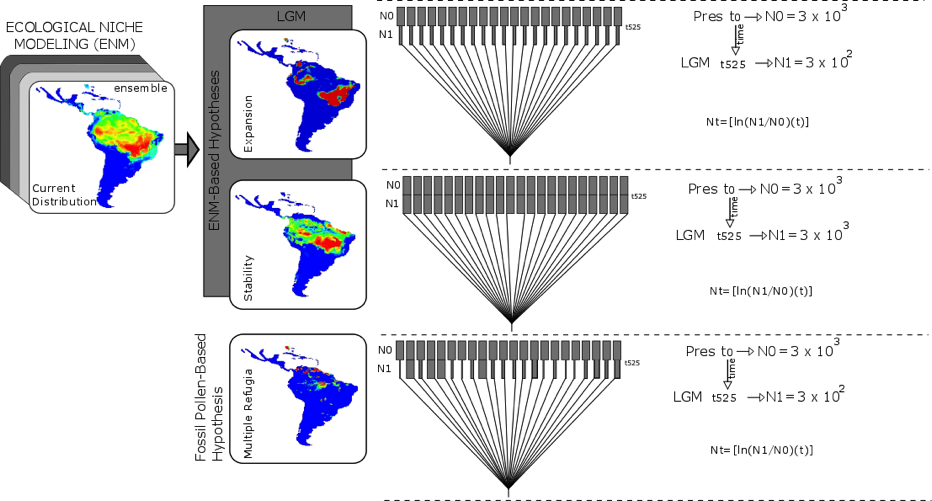
**

**Figure S1**. Demographic scenarios simulated for *Mauritia flexuosa* using the software DIYABC 2.1.0 and their geographical representation. The simulations were performed for 22 demes. LGM last glacial maximum, Pres present-day, N0 effective population size at time t0 (present), N1 effective population size at time t525 (525 generations ago corresponding to the LGM, 21 ka).

**
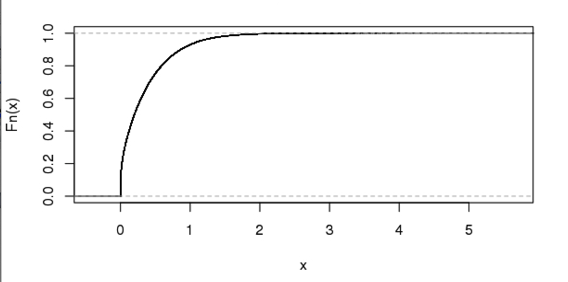
**

**Figure S2.** Cumulative histogram of composite likelihood simulated for neutral data sets across all positions along the length of the simulated sequences totalling 27 million of data points.

**
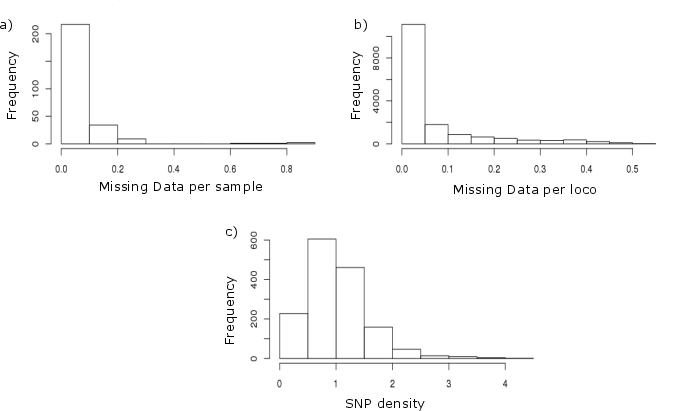
**

**Figure S3**. Missing data distributions for individuals, locus and density of SNPs for *Mauritia flexuosa* obtained using VCFtools. a) Percentage of missing data per individual, b) Percentage of data missing by loco, c) Density of SNPs using a bin size of 10 kbp.

**
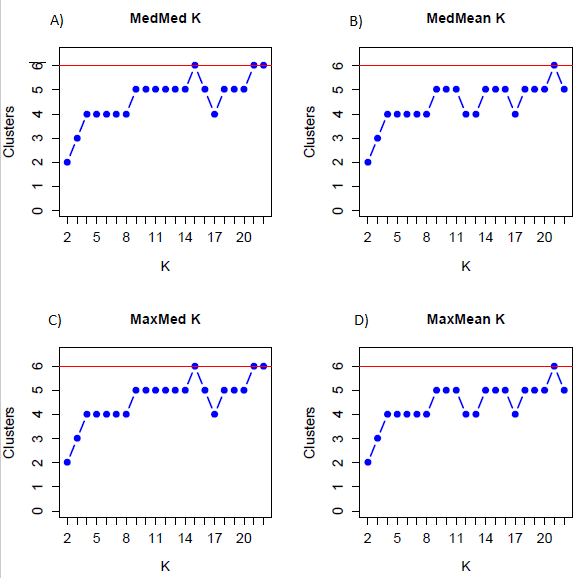
**

**Figure S4.** The most likely number of genetic groups (K = 6) for the 22 populations of *Mauritia flexuosa* based on four estimators (ΔK, corrected posterior probability (PP): (A) MedMedK’ (median of medians), (B) ‘MedMeanK’ (median of means), (C) Max-MedK’ (maximum of medians), and (D) ‘MaxMeanK’ (maximum of means). The best K is indicated by the red line.

‘

**
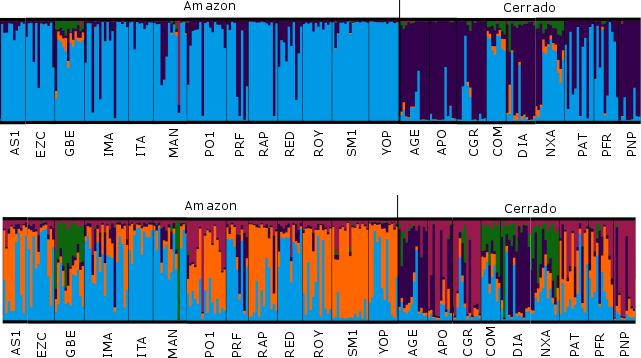
**

**Figure S5**. Composition of minor partitions (Minor modes) identified for 260 individuals from 22 *Mauritia flexuosa* populations. Each K value was executed 10 times and minor partition represented 2 in 10. For population codes refer to Supporting Information S1 Table S1. Each cluster is represented by a different colour. A threshold of 0.5 was used.


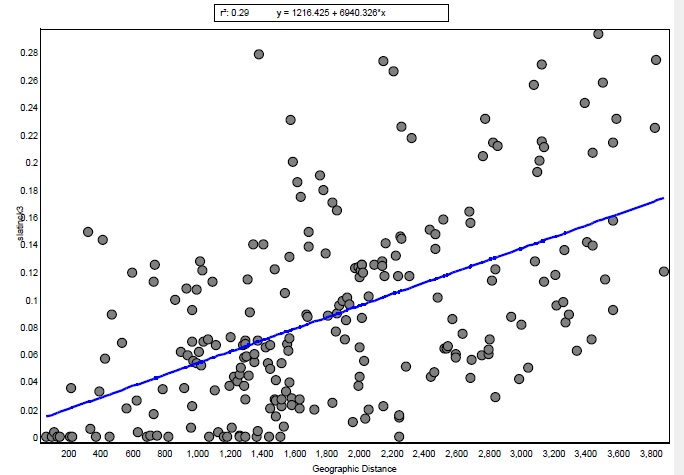


(**A**)

**
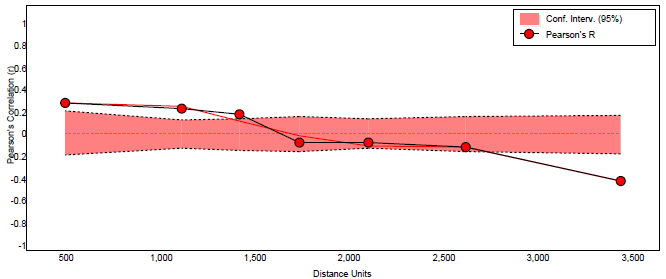
**

(**B**)

**Figure S6.** Relationship between genetic differentiation and geographical distance for *Mauritia flexuosa*, based on potentially neutral loci. (A) Regression of linearized *F_ST_* on geodesic geographical distance (logarithm) among all pair of populations. (B) Autocorrelation analysis based on Moran’s I for distance classes. For 500 km, r^2^ = 0.278, p < 0.001; 1000 km, r^2^ = 0.226, p = 0.003; 1500 km, r^2^ = 0.177, p = 0.025.


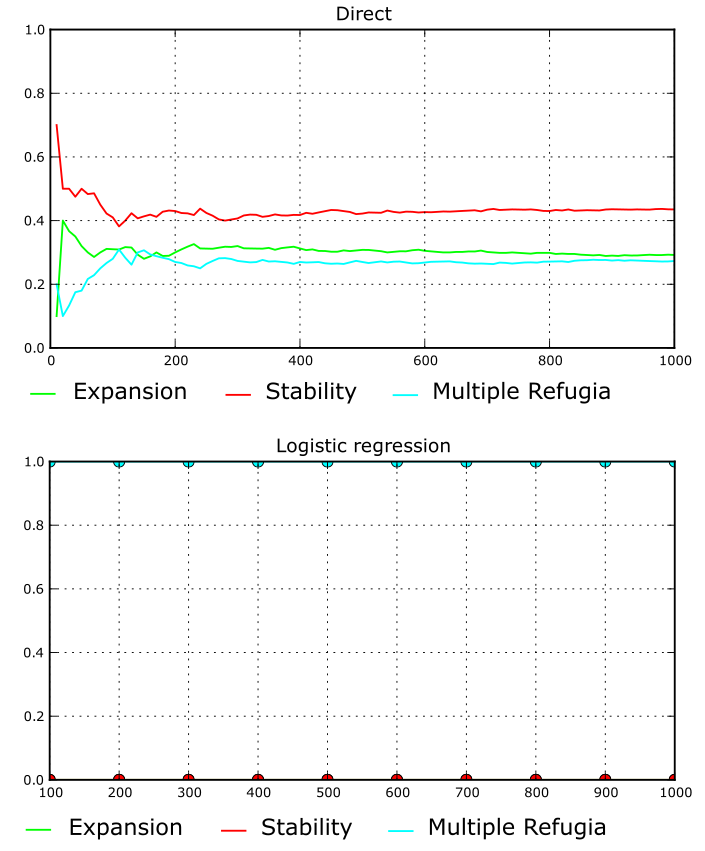


**Figure S7.** Posterior probabilities of demographic scenarios for populations *of Mauritia flexuosa* using Approximate Bayesian Computation (ABC).


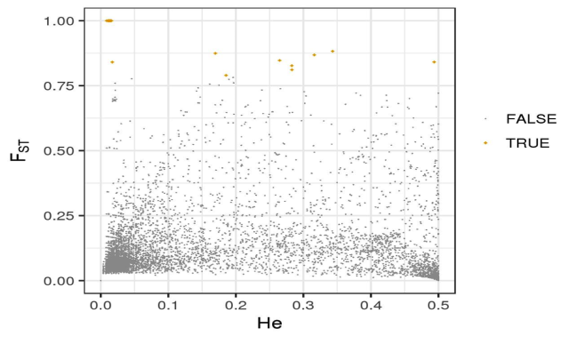


**Figure S8**. Plot of heterozygosity versus *F_ST_*. Yellow dots represent the 45 outlier SNPs identified by OutFLANK. Trim of 0.05.


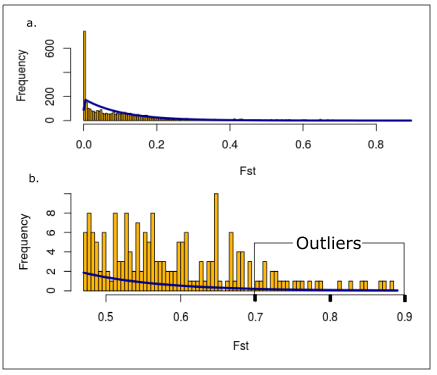


**Figure S9**. (a) *F_ST_* distribution of neutral loci estimated using OutFLANK method. (b) Distribution of *F_ST_* values above 0.5. Values equal to and greater than 0.7 are considered outliers.


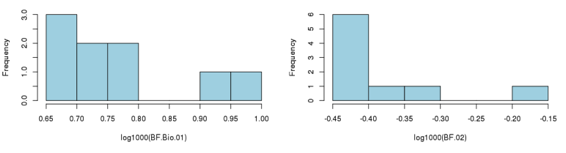


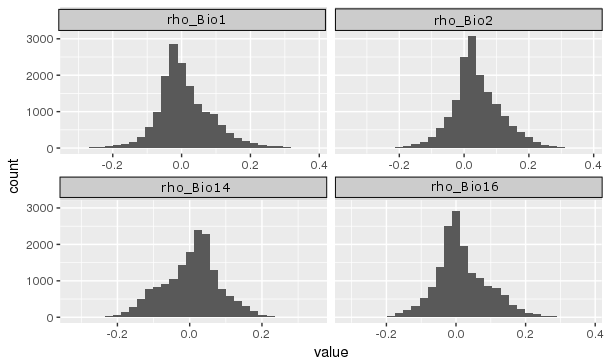


**Figure S10.** Frequency distribution of Bayes factor (BF) and Spearman’s correlation (rho) based on 16,262 SNPs of *Mauritia flexuosa* analysed with Bayenv2 software for climatic variables. Bio1, annual mean temperature; Bio2, mean diurnal range; Bio14, precipitation of driest month; Bio16, precipitation of the wettest quarter.


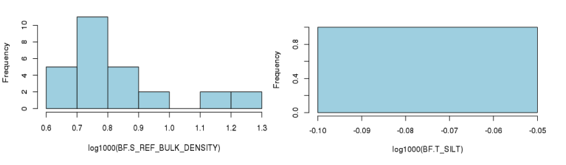


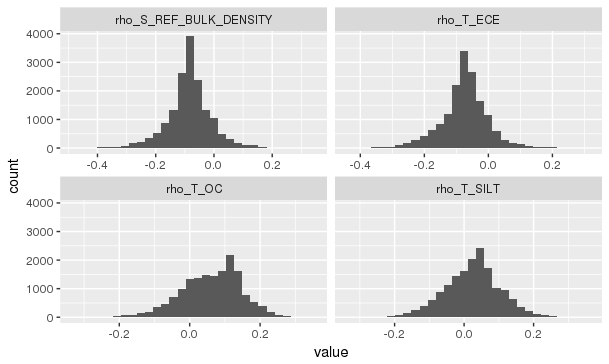


**Figure S11.** Frequency distribution of Bayes factor (BF) and Spearman’s correlation (rho) based on 16,262 SNPs of *Mauritia flexuosa* analysed with Bayenv2 software for soil variables. T_ECE, Topsoil Salinity (Elco); T_REF_BULK_DENSITY, Topsoil Reference Bulk Density; T_OC, Topsoil Organic Carbon; T_SILT, Topsoil Silt Fraction.


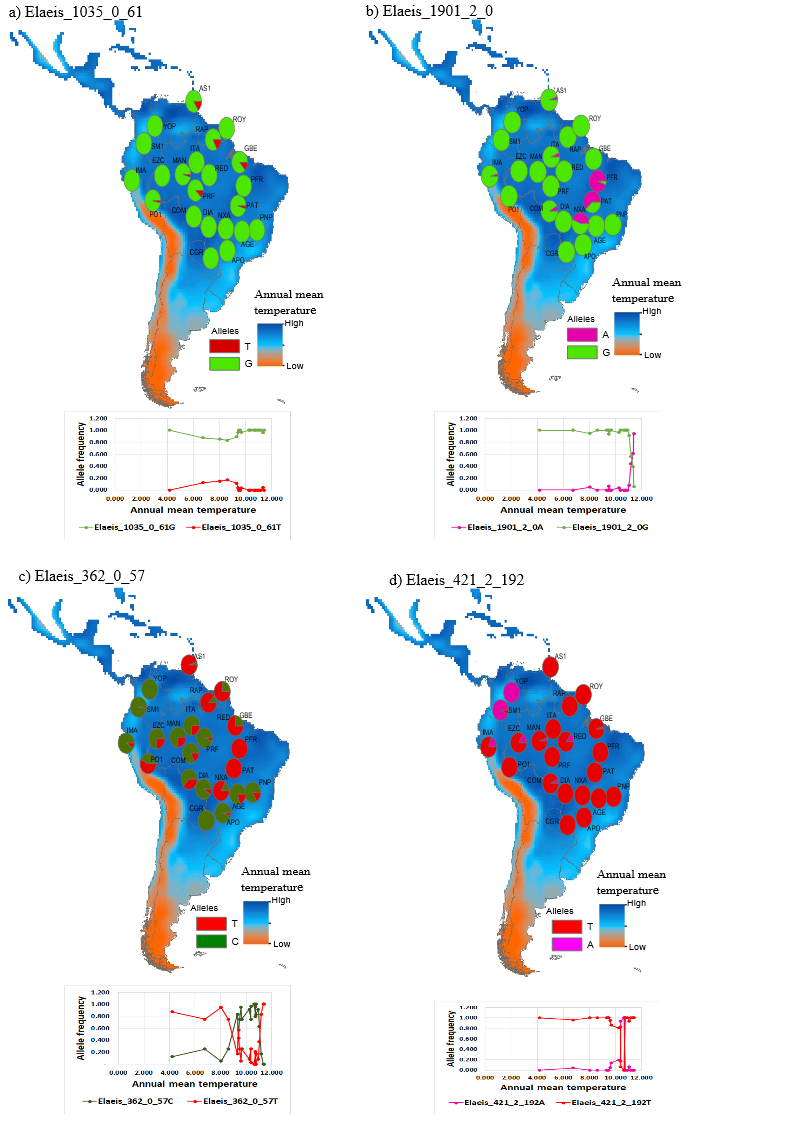


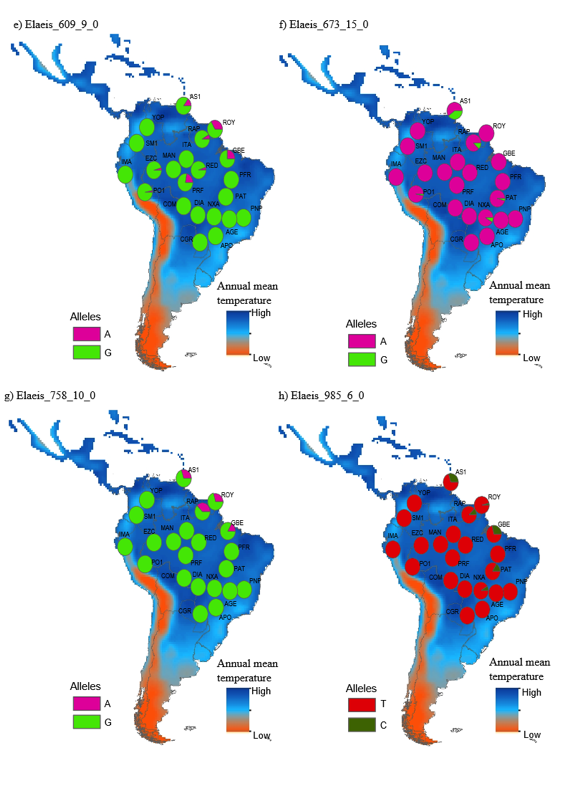


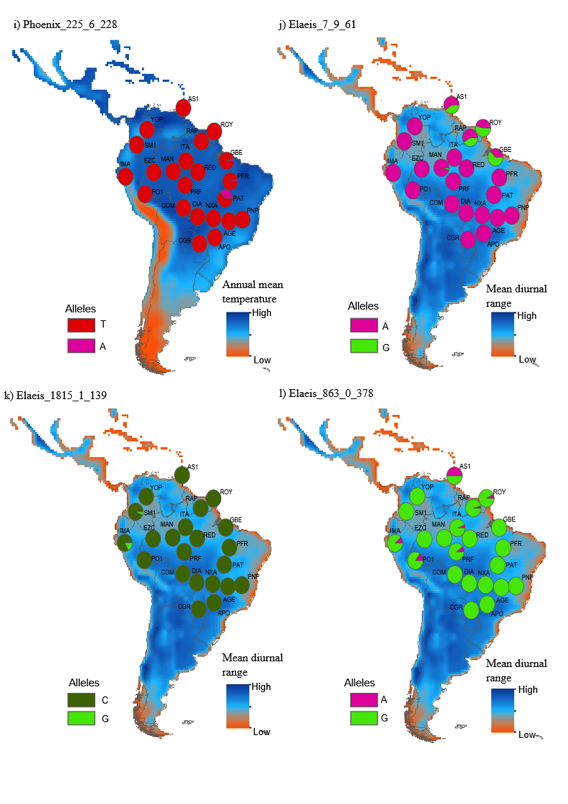


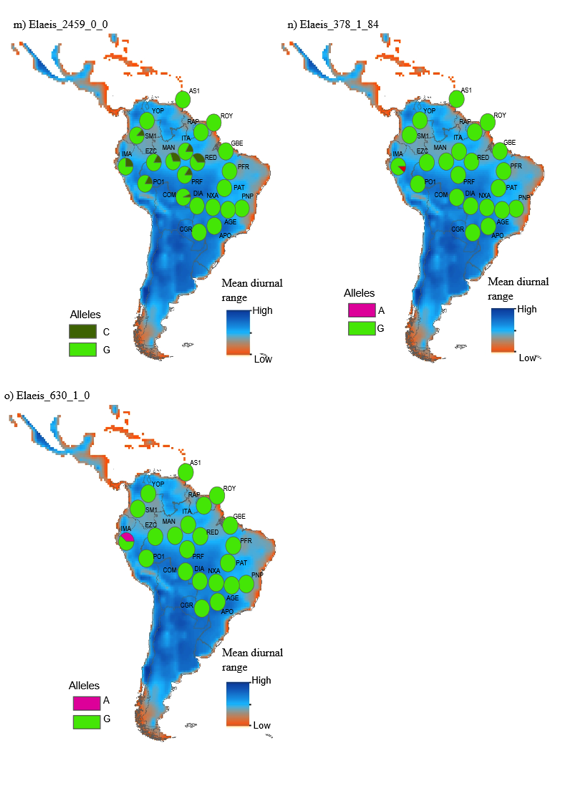


**Figure S12**. Geographical distribution of allele frequencies of loci with adaptive selection signal based on correlation of allele frequency and climatic variables. For details of the loci see Table S8 in Additional File S1.


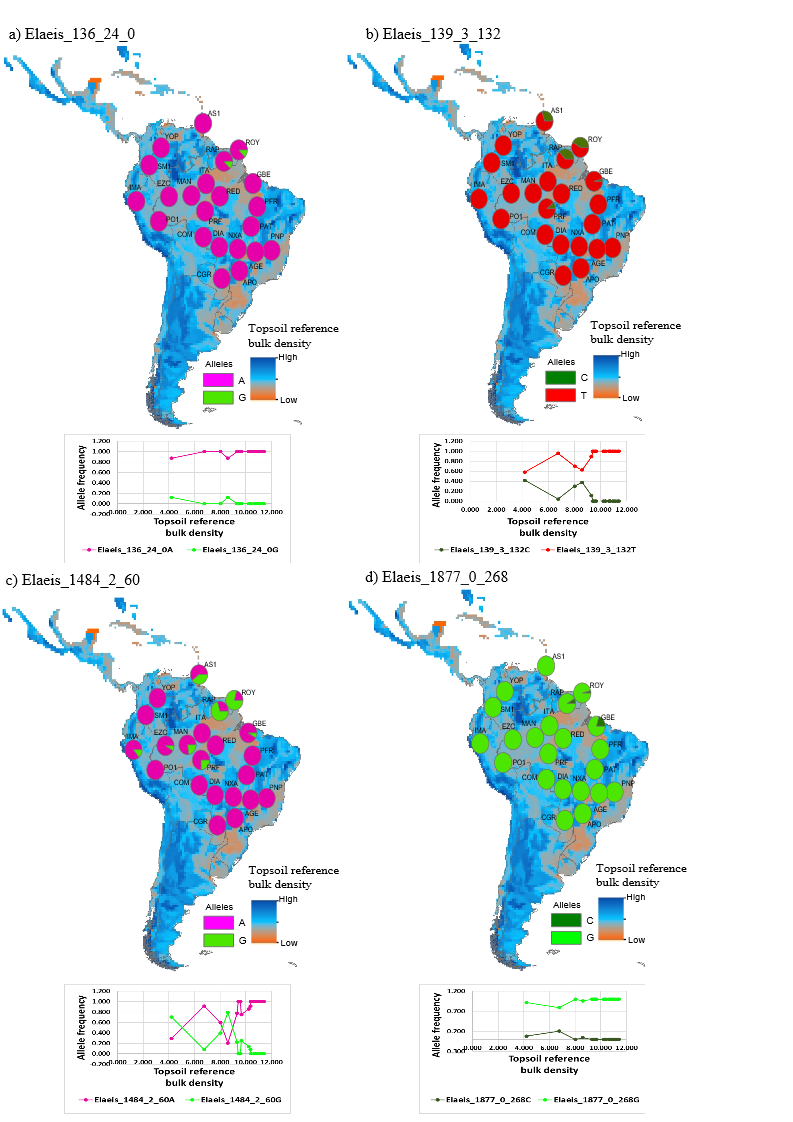


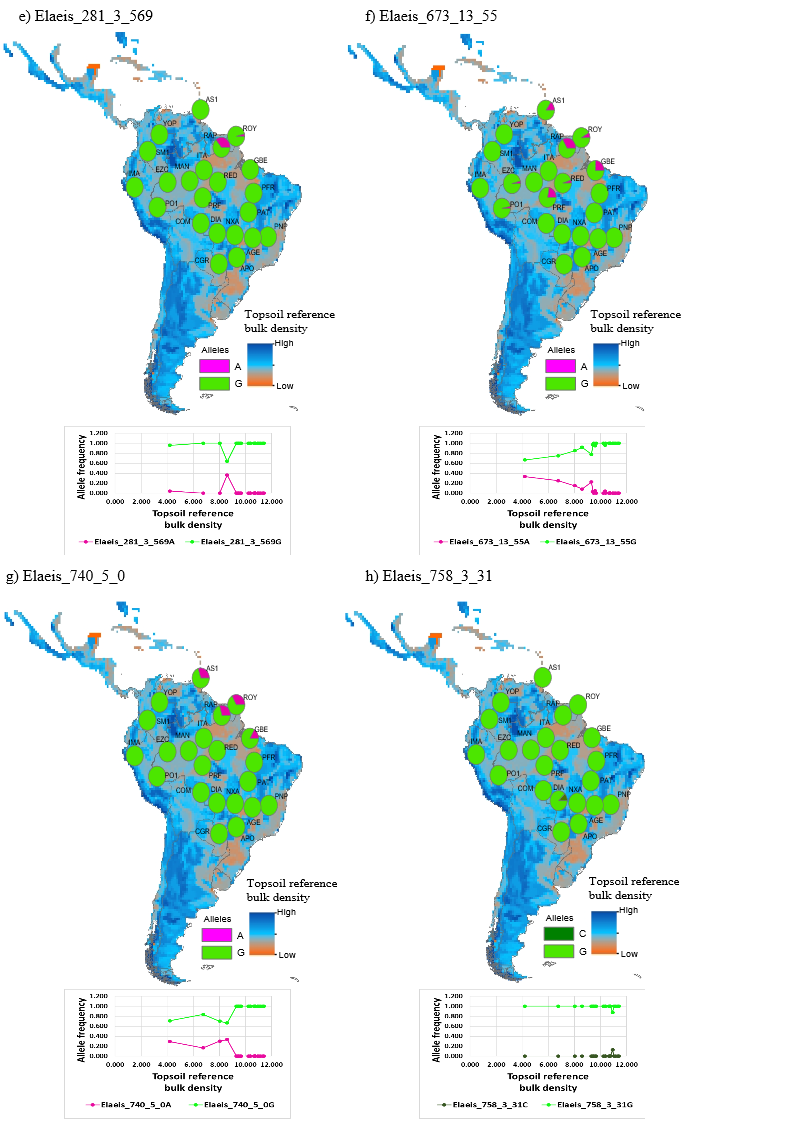


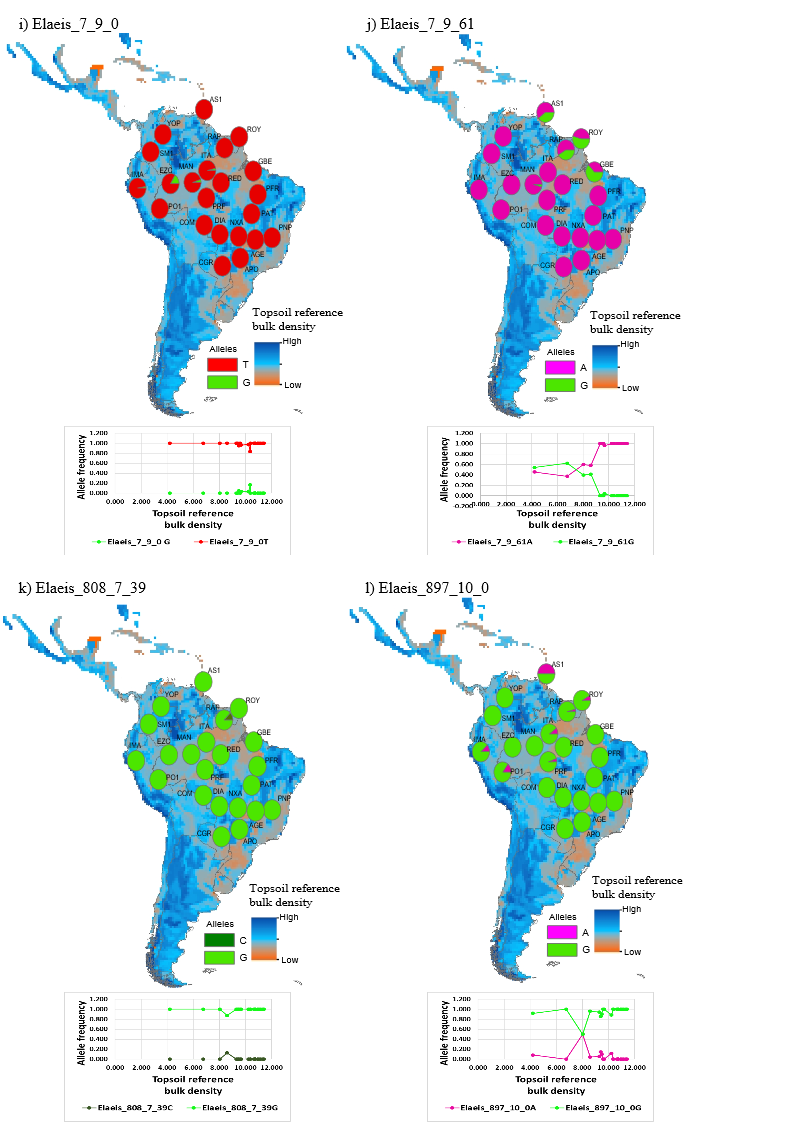


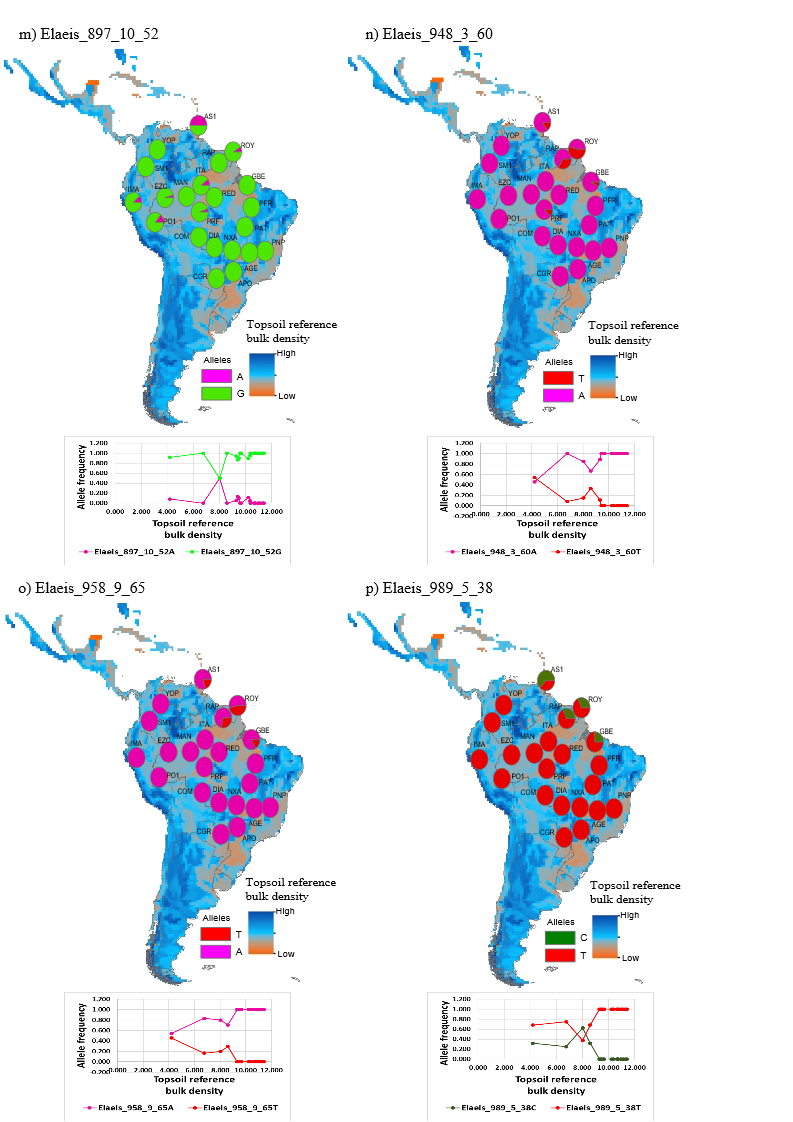


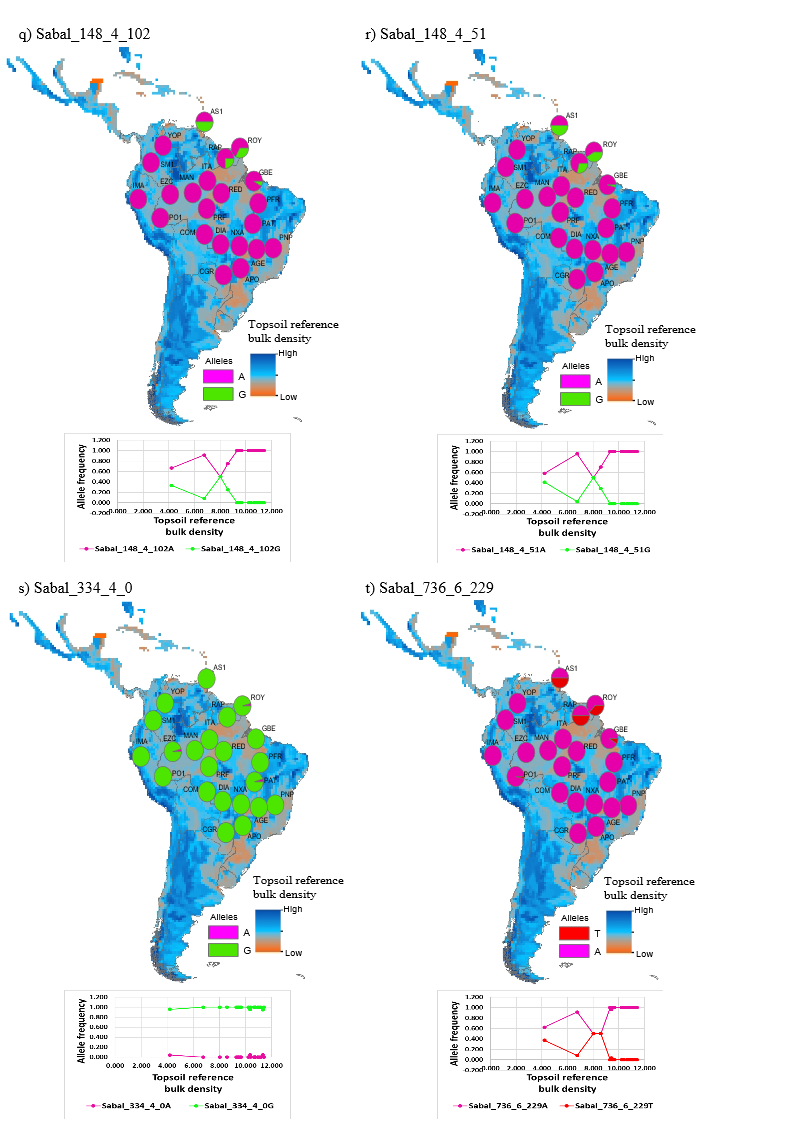


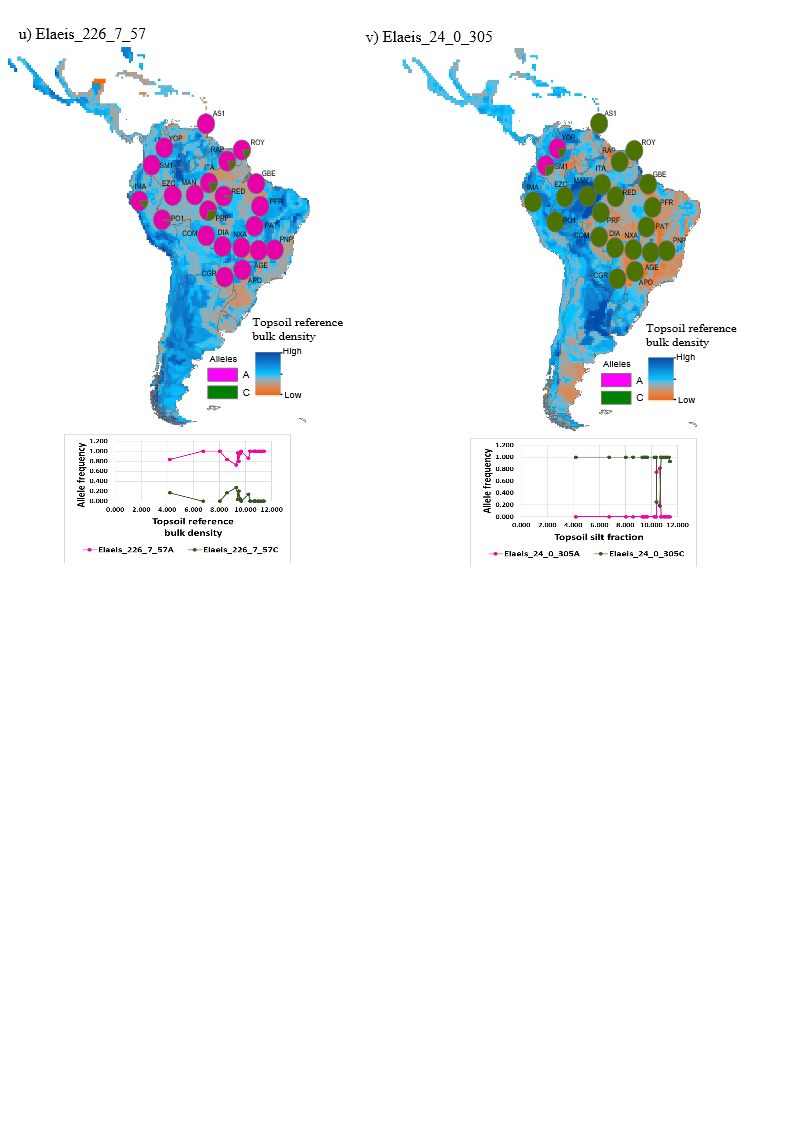


**Figure S13**. Geographical distribution of allele frequencies of loci with adaptive selection signal based on correlation of allele frequency and soil variables. For details of the loci see Table S9 in Additional File S1.
